# Facial skin aging: an integrative analysis of genetics, epigenetics, and lifestyle factors

**DOI:** 10.1007/s11357-025-02067-w

**Published:** 2025-12-24

**Authors:** Rezvan Noroozi, Aleksandra Pisarek-Pacek, Bożena Wysocka, Kamila Migacz-Gruszka, Paulina Pruszkowska-Przybylska, Magdalena Kobus, Dagmara Lisman, Julia Zacharczuk, Joanna Rudnicka, Aleksandra Iljin, Kathryn C. Fitzgerald, Małgorzata Michalczyk, Piotr Kaczka, Michał Krzysztofik, Maciej Kostrzewa, Aneta Sitek, Magdalena Spólnicka, Andrzej Ossowski, Wojciech Branicki, Ewelina Pośpiech

**Affiliations:** 1 Department of Neurology, School of Medicine, Johns Hopkins University, Baltimore, MD USA; 2 Malopolska Centre of Biotechnology, Jagiellonian University, Krakow, Poland; 3 Institute of Zoology and Biomedical Research of the Jagiellonian University, Krakow, Poland; 4 Central Forensic Laboratory of the Police, Warsaw, Poland; 5 Department of Dermatology, Jagiellonian University Medical College, Krakow, Poland; 6 Department of Anthropology, University of Lodz, Lodz, Poland; 7 Institute of Biological Sciences, Faculty of Biology and Environmental Sciences, Cardinal Stefan Wyszynski University in Warsaw, Warsaw, Poland; 8 Department of Genomics and Forensic Genetics, Pomeranian Medical University in Szczecin, Szczecin, Poland; 9 Doctoral School of Exact and Natural Sciences, Jagiellonian University, Krakow, Poland; 10 Department of Plastic, Reconstructive and Aesthetic Surgery, Medical University of Lodz, Lodz, Poland; 11 Institute of Sport Science, The Jerzy Kukuczka Academy of Physical Education in Katowice, Katowice, Poland; 12 Center for Forensic Science, University of Warsaw, Warsaw, Poland; 13 Regional Center of Digital Medicine, Pomeranian Medical University in Szczecin, Szczecin, Poland; 14 Institute of Forensic Research, Krakow, Poland; 15 Oncology Clinical Research Support Center, Polish Mother’s Memorial Hospital - Research Institute, Lodz, Poland

**Keywords:** Skin aging, Facial wrinkling, Perceived facial age, Lifestyle, DNA methylation biomarkers, Epigenetic age, MeQTL, *EDAR*, Anti-aging strategies, Genomic predictive modelling

## Abstract

Facial wrinkling is a prominent sign of aging, yet individuals exhibit unique trajectories of biological aging, contributing to the variability in facial appearance. Here, we present a pioneering study exploring the association between lifestyle choices, DNA methylation, and SNP genotypes with a range of facial skin aging phenotypes. The study demonstrated that age-related facial skin phenotypes are influenced by multiple environmental stressors. Epigenome-wide association analyses identified differentially methylated cytosines mapped to 151 *loci*, including novel genes associated with facial wrinkles, such as *EDAR* (cg02925966, *p* = 4.96 × 10^−8^) and *NRG1* (cg26267340, *p* = 4.64 × 10^−8^), both involved in wound healing. Sensitivity analysis, conditioning on the top meQTLs, showed minor attenuation, suggesting an association independent of genetic variants. Accelerated epigenetic aging, measured in the blood of over 700 Polish individuals, was found to correlate with facial wrinkle area, photoaging, telangiectasias, and perceived facial age, with the GrimAge and FitAge clocks showing the most robust associations. Genome-wide SNP analysis identified rs73943403 (RP11-78F17.1, *p* = 3.72 × 10^−8^) associated with wrinkle area and rs113125564 (*ZC3H4*, *p* = 1.23 × 10^−8^) associated with perceived facial age, potentially implicating stress response and adiposity in skin aging. Finally, the study revealed the additive value of methylation and SNP data for predicting two continuous skin phenotypes, with 59.0% of the variation explained in facial wrinkle area and 26.2% in perceived facial age. The findings underscore the relevance of genetic and epigenetic factors, revealing novel candidate genes and molecular pathways involved in skin aging. These insights hold substantial translational value for dermatology, the cosmetics industry, and anti-aging strategies.

**Supplementary Information:**

The online version contains supplementary material available at https://doi.org/10.1007/s11357-025-02067-w.

## Introduction

Skin condition is recognized as a reliable indicator of biological aging. This is supported by several studies showing that facial skin appearance is correlated with physiological dysregulation, age-related diseases, and mortality [1–5]. Therefore, studying the molecular basis of skin aging may offer insights into the underlying mechanisms of aging [6].

Progress in the study of human aging demands accurate clinical indicators of aging. Advances in omics technologies and access to large biobanks have been instrumental in the development of recent biological age clocks [7]. Accumulating evidence has demonstrated a strong link between aging and epigenetic alterations. DNA methylation–based age clocks have been developed, which are computational models for predicting epigenetic age by regressing chronological age and clinical biomarkers of aging on the methylation levels of selected sets of CpG sites [8–12]. Epigenetic age acceleration (EAA), which measures the discrepancy between chronological age and epigenetic age, has been associated with various health conditions and lifestyle factors and can be used to track the effectiveness of rejuvenating interventions [10, 13–15]. To some extent, EAA is inherited through DNA sequence variation, while the remaining variation in epigenetic aging is attributed to environmental factors [16]. Importantly, the correlation between differential methylation patterns and EAAs with facial skin traits has been studied to a very limited extent and has mostly been restricted to perceived facial age [15, 17, 18].

Facial aging is a complex condition characterized by a gradual deterioration of skin functions. It encompasses several distinct features, including wrinkles, pigment spots, eyelid laxity, and telangiectasias. As the skin ages, it undergoes various cellular and molecular changes, and as a result, the texture of the skin changes, becoming less elastic, thinner, darker, and harder [19, 20]. Skin aging can be influenced by genetics and other intrinsic factors such as hormone levels. The environmental factors linked so far include sun exposure, pollutants, smoking, body mass index (BMI), and alcohol consumption [21]. The heritability of skin wrinkles has been estimated to be ∼50% [22], and several genes have been implicated in skin aging, mainly involved in collagen synthesis, antioxidant defense, and DNA repair [23, 24]. However, the genetic basis of skin aging has not been rigorously investigated. Importantly, epigenetic modifications are increasingly emphasized as fundamental mechanisms that can further exacerbate the skin aging process by affecting gene expression [25, 26].

Research into the genes that control the pace of changes in facial skin can aid in identifying potential molecular targets for anti-aging treatments. This may be useful in dermatology and the cosmetic industry to maintain a youthful appearance [27]. Finally, these studies may have practical applications in forensic science. Predicting facial wrinkling and facial age can serve as clues in cold cases with no suspects or can assist in craniofacial reconstruction, thereby speeding up the process of investigation and personal identification [28–31].

In this study, we used age-correlated facial skin traits as a model to explore the molecular aspects of aging. We examined the association between lifestyle factors, DNA methylation, various epigenetic aging measures, and genetic variants with several skin aging phenotypes. The study materials included blood samples, detailed lifestyle data, and high-resolution 2D digital facial photographs and 3D facial scans from 735 Polish individuals. We identified a significant number of differentially methylated positions (DMPs) and novel single nucleotide polymorphisms (SNPs) associated with different facial skin aging traits. Finally, DNA methylation and SNP variants were assessed for their potential to predict skin wrinkles and perceived facial age.

## Materials and methods

### Participants in the study

A total of 735 blood samples were collected from Polish volunteers (48.4% male) aged 20–81 years, with a mean (SD) age of 46.4 (14.7) years (see age histogram in Supplementary Fig. 1). Written informed consent was obtained from all participants, and the study was approved by the Bioethics Committee of the Jagiellonian University in Krakow (decision nos. 1072.6120.132.2018, 1072.6120.31.2025). Demographic characteristics and detailed lifestyle data were collected from each participant through questionnaires and in-person interviews. Lifestyle-related risk factors included body mass index (BMI), smoking, alcohol consumption, coffee consumption, self-reported physical activity, type of diet, level of sugar intake, self-reported sensitivity to stress, practice of yoga or meditation, sleep duration, frequency of tanning, and history of excessive sun exposure (see Supplementary Tables 1 and 2).

### Skin aging phenotyping methods

**Fig. 1:**
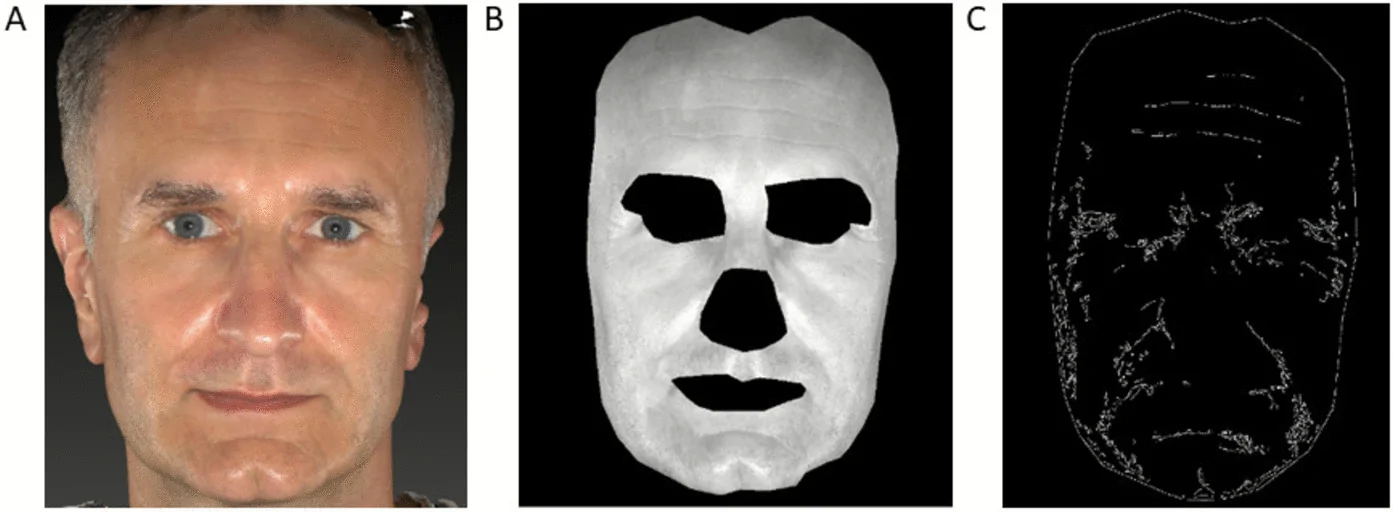
Semi-automated pipeline for facial wrinkle quantification and image preprocessing. **A** High-resolution 2D facial images were generated from standardized 3D face scans using VECTRA® H1 software (version 6.2). **B** Following preprocessing and contrast enhancement, a binary face mask was constructed to isolate the full-face skin area while excluding predefined regions of interest (ROIs) such as the eyes, eyebrows, nose, and mouth. A Gaussian smoothing filter was applied to reduce image noise and enhance surface continuity. **C** Wrinkle detection and quantification were performed using a Canny edge-detection algorithm implemented in MATLAB (https://www.mathworks.com). The output illustrates wrinkle mapping over the preprocessed face mask, representing the final pixels detected as wrinkles, which were subsequently used for the estimation of wrinkle area and wrinkle length in downstream analyses

Photonumerical classification and semi-automated computational quantitative methods were used to define various facial skin aging phenotypes. For all participants, 2D facial images (three angles including full face, right, and left profiles) and high-resolution 3D facial scans were obtained under optimized conditions with a standardized viewpoint, appropriate lighting, without makeup or jewelry using the VECTRA® H1 imaging system (Canfield Scientific, Inc.). Semi-automated facial wrinkle detection and quantification were done using MATLAB software (MATLAB (2018). 9.7.0.1190202 (R2019b). Natick, Massachusetts: The MathWorks Inc.). Wrinkles were detected using the Canny Edge Detector algorithm [32]. The detected wrinkles were quantified by calculating the facial wrinkle area, defined as the proportion of pixels identified as wrinkles relative to the total skin area (Fig. 1) [33]. Perceived facial age, subjective aging rate, Glogau classification of photoaging, photonumeric classification of full-face wrinkles, forehead wrinkles, crow’s feet wrinkles, lip wrinkles, eyelid drooping, nasolabial folds, pigmentation spots, telangiectasia, facial height, and lip height were also recorded in the study. Perceived facial age was determined by a certified dermatologist through visual assessment of each participant’s facial appearance, without prior knowledge of their chronological age [34]. Subjective aging rate was assessed on a 3-point scale, where participants self-reported their rate of aging: 0 = feeling younger than actual age, 1 = feeling the same as actual age, and 2 = feeling older than actual age. A detailed description of the phenotyping methods and the available sample numbers for each skin aging trait are provided in Supplementary Methods and Supplementary Table 3. To illustrate the trait distributions by sex, density plots were generated for quantitative variables and bar plots for categorical ones (see Supplementary Figs. 2 and 3).

For predictive modeling, two quantitative phenotypes were selected: wrinkle area and perceived facial age. Importantly, rather than using absolute values, the residuals from regressing these traits on age and sex, termed wrinkle area residuals and perceived facial age acceleration (see Supplementary Fig. 4), respectively, were used as inputs for the predictive analyses. Additionally, for some analyses, perceived facial age Δ was calculated as the simple difference between perceived age and chronological age.

### DNA methylation and epigenome-wide association analyses

The methylation profiles were successfully obtained for all 735 blood samples using the Illumina Infinium Methylation EPIC microarray (Illumina, San Diego, CA, USA). Primary quality control of the generated DNA methylation data was performed as previously described [35] and as summarized in the Supplementary Methods.

DMPs significantly associated (FDR *p* < 0.05) with specific skin phenotypes were identified through a series of epigenome-wide association (EWAS) analyses and using the limma package [36]. Linear models were fitted using derived methylation M-values, the log2 ratio of the intensities obtained from methylated probes to unmethylated probes. In fitted model 1, the results of the EWAS analysis were adjusted for age, sex, and cell components. Model 2 was additionally adjusted for environmental factors.

### Sensitivity analysis

Methylation quantitative trait loci (meQTLs) were identified through the analysis of single nucleotide polymorphisms (SNPs) located within ± 1 Mb of the top CpG sites associated with facial wrinkle area. A linear regression analysis was applied, with DNA methylation as the dependent variable and SNPs as the independent variable, adjusting the results for age and sex. Next, the top SNPs with the lowest *p* were used to rerun EWAS for wrinkle area, expanding the list of covariates to include the identified meQTLs, as suggested by [37].

### Profiling of epigenetic clocks and associations with facial skin aging

Epigenetic age was estimated for all *N* = 735 blood samples using the methylCIPHER R package across several clocks, including DNA methylation (DNAm) age Horvath, DNAm age Hannum, DNAm age Skin&Blood, DNAm PhenoAge, DNAm GrimAge, DNAm FitAge, and DNAmTL (see Supplementary Fig. 5 for the distribution of epigenetic ages) [8–12, 38, 39]. The methylation level, represented as the *β* value, was used as the input. The corresponding epigenetic age acceleration (EAA) measures were calculated as the residuals of regressing DNA methylation age on chronological age (Supplementary Methods). In addition, DNA methylation–based pace of aging (PACE and PoAm) and epigenetic Mortality Risk Score (Zhang MRS) were measured [14, 15, 40]. The EAA measures were next tested for association with different skin aging phenotypes. Model 1 was adjusted for sex and age, and model 2 was additionally adjusted for smoking status, stress level, skin color, frequency of tanning or solarium use, and blood cell proportions.

### Genome-wide association analyses

The Global Screening Array (GSA) from Illumina (San Diego, CA) was used for sample genotyping. Quality control prior to imputation was performed using PLINK1.9 [41], following the procedures described in [42] and in the Supplementary Methods. SNPs were then imputed using Beagle version 5.4 [43]. The final number of 477,827 SNPs and 719 samples was used for genome-wide association analyses (GWAS) performed using linear regression for testing genetic association with continuous skin aging phenotypes and logistic regression for binary skin aging traits under an additive genetic model. Multivariate regression models were adjusted for age, sex, BMI, skin color, smoking status, stress level, solarium exposure, and tanning frequency, and a *p*-value threshold of < 5 × 10^–8^ was considered statistically significant.

### Gene and pathway enrichment analysis

The missMethyl R package was applied to explore associations between differentially methylated CpGs and Gene Ontology (GO) terms [44, 45]. Genome-wide significantly associated SNPs were subjected to in silico functional analysis using HaploReg v4.2 [46] by exploring the annotation of DNA variants on haplotype blocks using linkage disequilibrium (LD) information from the 1000 Genomes Project [47], along with annotation from the Roadmap Epigenomics and ENCODE projects [48].

### Predictive analysis with LASSO

LASSO penalized regression, implemented in the glmnet R package [49], was employed to evaluate the predictive power of genetic and epigenetic data for two selected quantitative skin aging phenotypes: wrinkle area and perceived facial aging. Since both phenotypes are strongly associated with age, residuals from regressing the facial skin aging phenotypes on age and sex (yielding perceived facial age acceleration and wrinkle area residuals) were used as outcomes to avoid confounding effects, as described in the *Skin aging phenotyping methods* section. All CpGs (866,234) and/or all SNPs (477,827) were used as predictors. Missing CpG values were imputed using the mean value approach. Different models were compared based on the R^2^ and mean absolute error (MAE) parameters. The analysis was performed on the entire sample set, with tenfold cross-validation applied (with the mixing parameter alpha set to 1), extracting the best-fit lambda penalty values, when the error is minimized.

## Results

### Demographics, lifestyle, and facial skin aging

Age-adjusted male sex was significantly associated with accelerated facial skin aging in our study, including increased wrinkle area (Standardized B = 0.1, *p* = 7.6 × 10^–9^), higher perceived facial age (Std.B = 0.12, *p* = 1.03 × 10^–14^), drooping eyelids (OR = 1.87, 95% CI = 1.27, 2.75, *p* = 0.002), and Glogau classification of photoaging (OR = 3.29, CI = 1.87,5.96, *p* = 5.2 × 10^–5^). While higher levels of forehead (*p* = 6.4 × 10^–13^) and crow’s feet wrinkles (*p* = 0.04) were observed compared to women, men tended to have lower levels of wrinkles over the lips (*p* = 1.5 × 10^–4^, Supplementary Table 4).

Holding a university degree and high/medium socioeconomic status were significantly associated with subjectively scored slower rate of aging (OR = 0.64, CI = 0.46, 0.89, *p* = 0.007 and OR = 0.27, CI = 0.13, 0.56, *p* = 4.19 × 10^–4^, respectively). Importantly, individuals with higher education were also perceived as younger by others (perceived facial age, Std.B = −0.06, *p* = 2.32 × 10^–4^), and they had reduced levels of eyelid drooping (OR = 0.54, CI = 0.37, 0.80, *p* = 0.002) and reduced photoaging (OR = 0.38, CI = 0.21, 0.67, *p* = 0.0009). Consistently, individuals living in a city (> 100,000 population) had a slower rate of subjective aging (OR = 0.36, CI = 0.23, 0.57, *p* = 1.77 × 10^–5^) compared to those living in villages.

Frequent tanning was associated with facial perceived age and nasolabial folds (Std.B = 0.04, *p* = 0.009 and OR = 1.80, CI = 1.19, 2.75, *p* = 0.006, respectively). A significant association was found between current smoking and photonumeric (categorical) measurement of full-face wrinkles (OR = 6.30, CI = 1.55, 26.1, *p* = 0.009). Importantly, the effect size of wrinkles was greater in current smokers compared to former smokers (OR = 4.13, CI = 1.41, 13.3, *p* = 0.01). Stress caused participants to appear older than their chronological age (facial perceived age, Std.B = 0.05, *p* = 0.002) and led to more crow’s feet (OR = 3.04, CI = 1.47, 6.29, *p* = 0.003). Physical activity reduced photoaging (OR = 0.50, CI = 0.28, 0.91, p = 0.02). Higher BMI was found to be associated with increased telangiectasias (OR = 1.09, CI = 1.05, 1.14, *p* = 7.74 × 10^–6^) and eyelid drooping (OR = 1.12, CI = 1.07, 1.17, *p* = 4.01 × 10^–7^). However, higher BMI decreased subjectively assessed rate of aging (OR = 0.11, CI = 1.07, 1.15, *p* = 2.80 × 10^–7^), and participants also reported feeling younger when they exercised (*p* = 0.01), did yoga (*p* = 4.30 × 10^–5^), and followed a vegetarian diet (*p* = 0.008). Interestingly, practicing yoga was also associated with reduced levels of crow’s feet wrinkles (*p* = 0.03). Sugar consumption was associated with drooping eyelids in our study (OR = 3.19, CI = 1.43, 7.83, *p* = 0.007). Detailed results are reported in Supplementary Table 4.

### EWAS identified novel blood DNA methylation markers for facial skin aging

At the FDR-corrected *p*-value (adj. *p* < 0.05), significant DMPs were detected in model 1, which corrects for age, sex, and cell components, for the following traits: wrinkle area (3 CpGs), wrinkle length (5 CpGs), photonumeric full-face wrinkle score (64 CpGs), and lip wrinkles (158 CpGs) (Supplementary Table 5). The results remained significant (or near significant) in the vast majority of cases also in model 2, when correction for lifestyle choices was applied (Supplementary Table 6). Table 1 shows the list of the top significant DMPs. The highest significance for wrinkle area was detected for cg02925966 in *EDAR* (raw *p* = 1.14 × 10^−7^), while two other highly significant ones, cg26267340 in *NRG1* and cg27278787 in *DLGAP1*, were also associated with lip wrinkles (adj. *p* < 0.05).

**Table 1:** Top significant differentially methylated positions (DMPs) associated with selected facial skin aging traits, identified through EWAS analyses adjusted for age, sex, cell composition, and lifestyle factors

Facial skin aging trait	CpG ID	Chr	hg19	UCSC Ref. gene	logFC	Ave. Meth.	*p*	adj. *p*
**Wrinkle area**	cg02925966	2	109552331	*EDAR*	−3.711	1.713	1.14 × 10^–7^	0.04
	cg26267340	8	31497829	*NRG1*	−4.122	−0.672	1.54 × 10^–7^	0.04
	cg27278787	18	3499813	*DLGAP1*	6.330	−1.447	1.61 × 10^–7^	0.04
**Photonumeric full-face wrinkle score**	cg06513979	10	617105	*DIP2C*	−0.438	0.846	8.14 × 10^–9^	0.006
	cg10602219	8	142040925	NA	−0.279	−1.014	4.95 × 10^–8^	0.01
	cg03461391	10	1033524	*GTPBP4*	−0.386	−1.126	6.12 × 10^–8^	0.01
	cg06941230	16	11484506	NA	−0.221	1.308	1.04 × 10^–7^	0.01
	cg02964208	2	101618150	*RPL31*	0.260	0.186	1.12 × 10^–7^	0.01
	cg07579123	1	64323322	*ROR1*	−0.256	0.929	1.29 × 10^–7^	0.01
	cg17028912	1	11619369	NA	−0.272	1.428	1.36 × 10^–7^	0.01
	cg02551095	15	33597346	NA	−0.283	1.628	1.57 × 10^–7^	0.01
	cg26915329	13	60033345	NA	−0.213	−0.156	1.98 × 10^–7^	0.01
	cg27415945	20	1310451	*FKBP1A-SDCBP2*	−0.202	2.244	2.94 × 10^–7^	0.02
**Lip wrinkles**	cg08961558	17	1837648	*NA*	−0.412	2.257	4.13 × 10^–11^	3.09 × 10^–5^
	cg12484193	2	183943469	*DUSP19*	0.589	−4.424	8.95 × 10^–9^	0.003
	cg21854184	12	124870112	*NCOR2*	−0.264	2.407	1.04 × 10^–8^	0.003
	cg09423021	1	226960554	NA	−0.359	1.342	2.39 × 10^–8^	0.004
	cg14354398	13	113539759	*ATP11A*	−0.343	1.656	2.80 × 10^–8^	0.004
	cg17981552	4	8643813	NA	−0.293	0.498	3.49 × 10^–8^	0.004
	cg19673640	22	26860199	*HPS4*	−0.214	1.430	4.38 × 10^–8^	0.005
	cg20443138	2	183943496	*DUSP19*	0.484	−3.940	6.33 × 10^–8^	0.005
	cg01596963	11	62455830	*LRRN4CL*	−0.171	−0.027	6.74 × 10^–8^	0.005
	cg12712899	3	65583459	*MAGI1*	0.301	−1.949	7.06 × 10^–8^	0.005

*CpG ID*, the Illumina CpG identifier; *UCSC Ref. gene*, the gene name according to HUGO Gene Nomenclature Committee assigned to the CpG; *logFC (M-values)*, log₂-scaled estimate of the association between methylation level (*M*-values) and the tested phenotype; positive values indicate hypermethylation, i.e., higher methylation with increasing value in the phenotype; negative values indicate hypomethylation, i.e., lower methylation with increasing value in the phenotype; *Ave. Meth.*, mean log₂-transformed methylation (*M*-value) across all samples; *p*, unadjusted *p* obtained from regression analysis implemented in the limma package; *adj. p*, the adjusted *p*-value for multiple tests with FDR correction method with *p* < 0.05 cutoff point. The table presents the results of EWAS analyses using model 2, adjusted for age, sex, cell composition, and lifestyle factors. Wrinkle area was calculated from 3D facial scan analysis as the proportion of pixels identified as wrinkles relative to the total skin area, using the Canny Edge Detector algorithm [32]; photonumeric classification of full-face and lip wrinkles was performed by a certified dermatologist, with wrinkles defined in a binary manner as small (reference) or deep

### EWAS findings are largely independent on genetic variants

Potential confounding effects due to genetic variation were evaluated by first identifying methylation quantitative trait loci (meQTLs), followed by rerunning the EWAS for wrinkle area while adjusting for the top meQTLs. For the three top CpG sites identified in the EWAS, cg02925966 in *EDAR*, cg26267340 in *NRG1*, and cg27278787 in *DLGAP1*, significantly associated SNPs adjacent to the reported CpGs (after FDR correction) were identified for *NRG1* and *DLGAP1* (Supplementary Table 7). For *EDAR*, the top SNPs with raw *p* < 0.01 were used as meQTLs. Conditioning the analysis on the top meQTLs led to only a slight reduction in the association signal, suggesting the DNA methylation effect is predominantly independent of genetic variation (Supplementary Table 8). The influence of genetic variants on the association between DNA methylation and wrinkle area was least pronounced for *EDAR* (EWAS *p* = 9.37 × 10^–8^).

### Facial skin aging associates with epigenetic age acceleration

Of all the epigenetic clocks used, GrimAge showed the most consistent association with various facial skin aging characteristics. Accelerated GrimAge was associated with a larger facial wrinkle area (Std.B = 0.07, *p* = 3.43 × 10^–4^), Glogau’s photoaging (OR = 1.08, CI = 1.01, 1.16, *p* = 0.03), the occurrence of lip wrinkles (OR = 1.13, CI = 1.01, 1.26, *p* = 0.03), telangiectasias (OR = 1.06, CI = 1.01, 1.1, *p* = 0.01), higher subjective aging (OR = 1.06, CI = 1.01, 1.10, *p* = 0.01), or older perceived age (Std.B = 0.06, *p* = 1.47 × 10^–4^). Most of these associations remained significant in a fully adjusted model, with the strongest associations observed for GrimAgeAccel and perceived age or wrinkle area (Table 2). Higher FitAge was associated with a larger facial wrinkle area (Std.B = 0.04, *p* = 0.02), higher perceived facial age (St.B = 0.05, *p* = 5.04 × 10^–4^), and a higher subjective rate of aging (OR = 1.06, CI = 1.01, 1.10, *p* = 0.008). Increased pace of aging, as measured by PoAm, showed an association with perceived facial age (Std.B = 0.05, *p* = 0.003), while an association with the subjective rate of aging was also reported using an extended version of the pace of aging parameter, i.e., PACE (OR = 5.12, CI = 1.24, 21.43, *p* = 0.02). The results of the associations with epigenetic aging for several selected skin aging traits are summarized in Table 2, while the findings for the other phenotypes are presented in Supplementary Table 9.

**Table 2:** Association between selected facial skin aging phenotypes and multiple epigenetic aging metrics in the studied population

Facial skin aging trait	Wrinkle area *N* = 735		Perceived facial age *N* = 649		Subjective aging rate *N* = 728			Pigmentation spots *N* = 735			Telangiectasias *N* = 735		
	Std.B	***p***	Std.B	***p***	OR	95% CI	***p***	OR	95% CI	***p***	OR	95% CI	***p***
**Model 1 (age and sex adjusted)**													
DNAm age Horvath	−0.05	0.33	−0.04	0.40	0.99	0.96, 1.02	0.54	1.01	0.96, 1.08	0.65	0.98	0.95, 1.01	0.26
DNAm age Hannum	0.07	0.25	0.03	0.57	0.99	0.95, 1.03	0.59	1	0.93, 1.07	0.99	1.01	0.97, 1.05	0.81
DNAm age Skin&Blood	−0.003	0.97	−0.05	0.45	0.98	0.94, 1.02	0.35	**1.12**	**1.03**, **1.21**	**0.007**	1.03	0.98, 1.07	0.28
IEAA	−0.02	0.27	−0.02	0.16	0.99	0.95, 1.02	0.43	1.01	0.95, 1.07	0.77	0.98	0.95, 1.02	0.26
EEAA	0.03	0.13	0.009	0.56	0.99	0.96, 1.02	0.55	0.99	0.94, 1.04	0.66	1.01	0.98, 1.04	0.61
PhenoAgeAccel	0.002	0.92	−0.002	0.89	0.98	0.95, 1.01	0.13	1.01	0.96, 1.06	0.8	**1.04**	**1.01**, **1.07**	**0.009**
GrimAgeAccel	**0.07**	**3.43 × 10**^**–4**^	**0.06**	**1.47 × 10**^**–4**^	**1.06**	**1.01**, **1.10**	**0.01**	1	0.93, 1.08	0.99	**1.06**	**1.01**, **1.10**	**0.01**
FitAgeAccel	**0.04**	**0.02**	**0.05**	**5.04 × 10**^**–4**^	**1.06**	**1.01**, **1.10**	**0.008**	1.03	0.96, 1.1	0.44	1.04	1.00, 1.08	0.08
PACE	0.02	0.27	0.01	0.43	**5.12**	**1.24**, **21.43**	**0.02**	1.48	0.12, 20.17	0.76	**13.33**	**3.02**, **59.57**	**0.001**
PoAm	0.03	0.12	**0.05**	**0.003**	4.20	0.55, 32.53	0.17	0.12	0, 4.18	0.23	**8.76**	**1.10**, **70.53**	**0.04**
Zhang MRS	**0.05**	**0.01**	0.03	0.06	0.95	0.61, 1.48	0.83	0.64	0.3, 1.41	0.27	**1.87**	**1.19**, **2.94**	**0.007**
DNAmTL	−0.04	0.29	−0.04	0.20	0.77	0.33, 1.81	0.55	0.31	0.06, 1.44	0.14	1.04	0.42, 2.53	0.94
**Model 2 (adjusted for age, sex, lifestyle and blood cell proportion)**													
IEAA	−0.01	0.52	−0.02	0.22	0.98	0.95, 1.02	0.30	1.01	0.95, 1.08	0.71	0.98	0.95, 1.02	0.29
EEAA	0.04	0.10	0.02	0.38	1.01	0.97, 1.04	0.78	1.01	0.95, 1.08	0.74	0.99	0.96, 1.03	0.78
Skin&BloodAgeAccel	0.01	0.59	−0.02	0.27	0.98	0.93, 1.03	0.38	**1.12**	**1.03**, **1.22**	**0.01**	1.03	0.98, 1.08	0.22
PhenoAgeAccel	−0.002	0.89	0.003	0.84	0.99	0.96, 1.02	0.49	1.03	0.98, 1.09	0.27	1.03	1.00, 1.06	0.07
GrimAgeAccel	**0.06**	**0.008**	**0.06**	**9.34 × 10**^**–4**^	**1.07**	**1.02**, **1.13**	**0.003**	1.07	0.98, 1.18	0.13	**1.06**	**1.01**, **1.11**	**0.03**
FitAgeAccel	0.03	0.06	**0.05**	**0.002**	**1.05**	**1.01**, **1.10**	**0.02**	1.07	0.99, 1.16	0.09	1.04	0.99, 1.09	0.08
PACE	0.01	0.52	0.01	0.44	**7.23**	**1.55**, **34.20**	**0.01**	4.73	0.3, 80.48	0.28	**12.94**	**2.59**, **65.61**	**0.002**
PoAm	0.01	0.56	**0.06**	**0.001**	9.46	0.87, 104.19	0.07	0.84	0.01, 61.89	0.94	7.25	0.63, 84.94	0.11
Zhang MRS	0.06	0.06	**0.06**	**0.01**	1.62	0.83, 3.20	0.15	1.46	0.46, 4.81	0.53	1.71	0.86, 3.41	0.12
DNAmTL	−0.05	0.20	−0.02	0.42	0.84	0.33, 2.16	0.72	0.20	0.03, 1.09	0.07	1.00	0.38, 2.68	0.99

Results for selected facial skin aging traits; other traits are shown in Supplementary Table 9

The analysis included selected epigenetic clocks, namely DNAm Age Horvath2013, DNAm Age Hannum, DNAm age Skin&Blood, and DNAmTL. Additionally, epigenetic age acceleration (EAA) measures were calculated as the residuals from regressing DNAm age on chronological age. EAA measures comprised intrinsic epigenetic age acceleration (IEAA), calculated using the Horvath2013 clock; extrinsic epigenetic age acceleration (EEAA), estimated using the Hannum clock; DNAm PhenoAge acceleration (PhenoAgeAccel); DNAm GrimAge acceleration (GrimAgeAccel); and DNAm FitAge acceleration (FitAgeAccel). Additionally, DNAm-based pace of aging metrics (PACE and PoAm) and the epigenetic mortality risk score (Zhang MRS) were included in the analysis

Wrinkle area and perceived facial age were measured on a continuous scale; the subjective aging rate, telangiectasias, and pigmentation spots were measured on a 3-point scale and analyzed using ordinal regression

*Std.B*, standardized beta coefficient from linear regression; *OR*, odds ratio from logistic regression; *95% CI*, 95% confidence interval

Model 2 for continuous traits: lm(wrinkle(linear) ~ EAA + Age + Sex + Smoking + Stress + Skin color Fitzpatrick + Solarium + Frequency of tanning + CD8T + CD4T + NK + Bcell + Mono + Gran)

Model 2 for binary traits: glm(wrinkle (binary) ~ EAA + Age + Sex + Smoking + Stress + Skin color Fitzpatrick + Solarium + Frequency of tanning + CD8T + CD4T + NK + Bcell + Mono + Gran)

Significant results are marked with bold

### Novel SNPs associated with facial wrinkling and perceived facial age

**Fig. 2:**
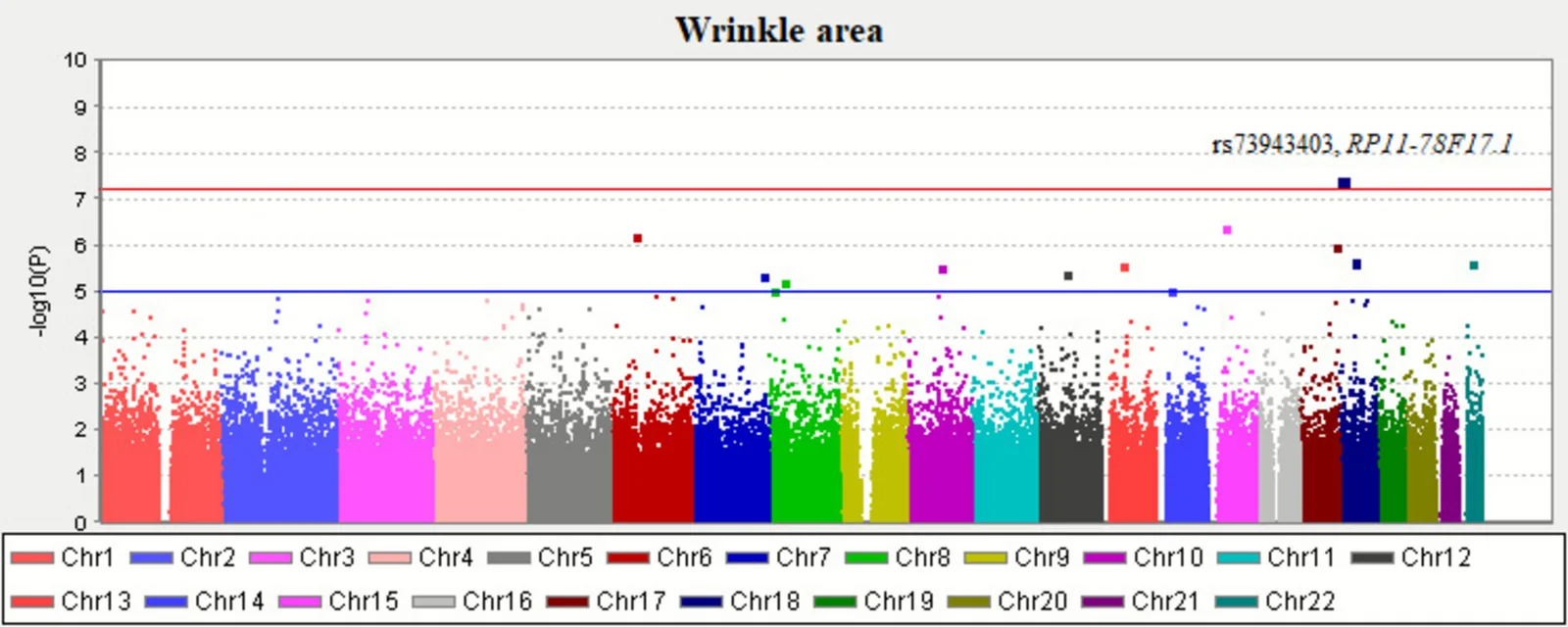
Manhattan plot for GWAS on wrinkle area. The rs73943403 is located on chromosome 18 (*p* = 3.72 × 10^–8^). The red line indicates the genome-wide significant level (*p* = 5 × 10^–8^), and the blue line indicates the suggestive *p*-value. The results were adjusted for age, sex, BMI, skin color, smoking status, stress level, solarium exposure, and tanning frequency

**Fig. 3:**
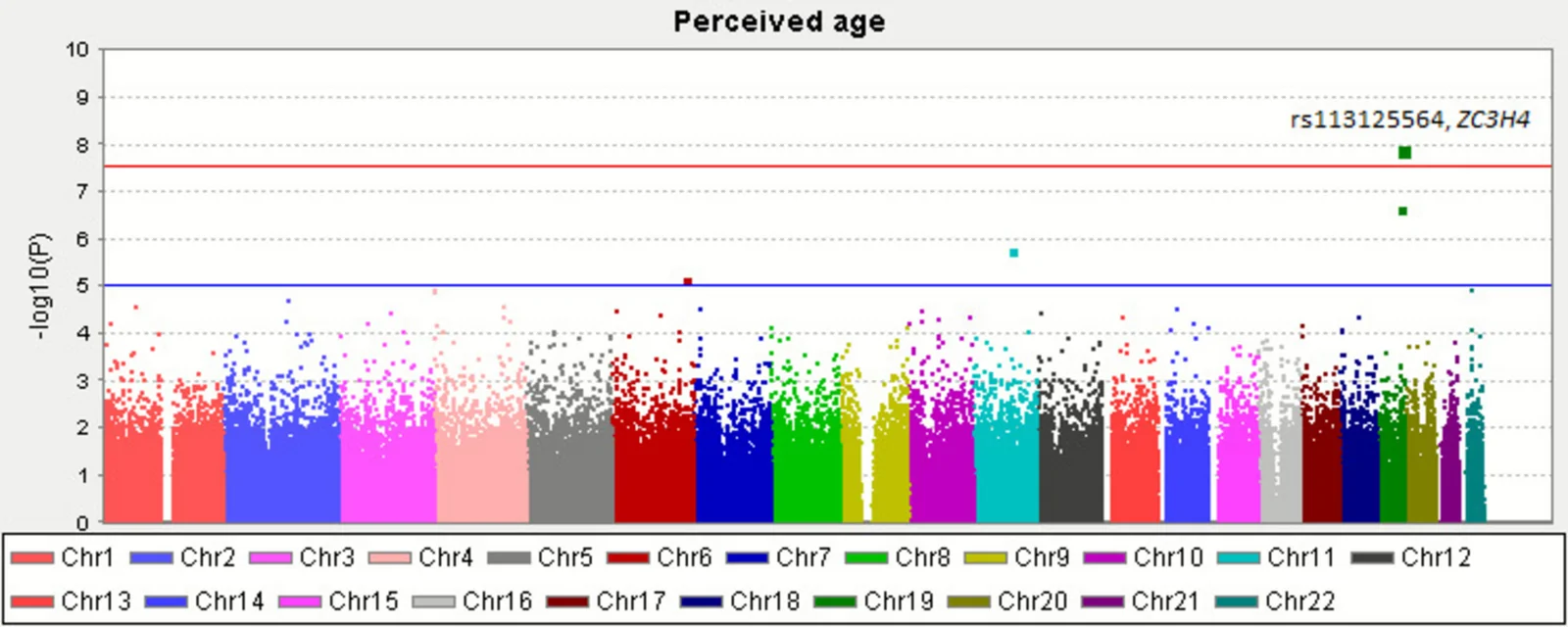
Manhattan plot for GWAS on perceived facial age. The rs113125564 located on chromosome 19 (*p* = 1.2 × 10^–8^). The red line indicates the genome-wide significant level (*p* = 5 × 10^–8^), and the blue line indicates the suggestive *p*-value. The results were adjusted for age, sex, BMI, skin color, smoking status, stress level, solarium exposure, and tanning frequency

A genome-wide significant association (*p* < 5 × 10^−8^) was found for facial wrinkle area with rs73943403 located on chromosome 18 and mapped to the *RP11-78F17.1* gene (*p* = 2.79 × 10^–8^, Fig. 2). Nearly significant associations with wrinkle area were also observed with rs79930775 in *TRAM2-AS1* (*p* = 2.42 × 10^–7^) and rs117218527 in *AQR* (*p* = 5.16 × 10^–7^). Genome-wide significance was found for perceived facial age associated with the intronic SNP, rs113125564, located on chromosome 19 and mapped to the *ZC3H4* gene (*p* = 2.85 × 10^–8^), as shown in Fig. 3, and a suggestive association was seen for rs45607935 in the *BBC3* gene (*p* = 5.26 × 10^–7^). Association close to significant was also observed for rs77826389 in *HMCN2* for wrinkle length (*p* = 8.87 × 10^–7^). All suggestive associations (*p* < 1 × 10^–5^) are listed in Supplementary Table 10.

### Pathways enrichment analysis

The reported DMPs were mapped through gene ontology (GO) enrichment analysis to 151 unique genes (Supplementary Table 5). Some of the top enriched GO terms were cell communication (*p* = 0.0008), spinal cord interneuron axon guidance (*p* = 6.95 × 10^–5^), nervous system development (*p* = 0.0003), regulation of collagen binding (*p* = 0.003), and RNA polymerase I cis-regulatory region sequence–specific DNA binding (*p* = 0.002, Supplementary Table 11). The functional analysis for the query SNP and variants in LD (R^2^ >  = 0.8) is reported in Supplementary Table 12.

### Machine learning for predictive analysis

Two continuous phenotypes, wrinkle area and perceived facial age, were selected for predictive analyses. Rather than the absolute values, regression residuals on age and sex were used, resulting in wrinkle area residuals and perceived facial age acceleration as the outcomes. The best models for wrinkle area identified 2 CpGs (including cg02925966 at *EDAR*) or 129 SNPs with non-zero weights when large-scale methylation and SNP data were analyzed, respectively. These selected markers, beyond age and sex, explained 1.1% and 39.5% of the variation in measured wrinkle area, as determined by the R^2^ parameter (Table 3). The best model for perceived facial age, based on genome-wide methylation data, returned 50 CpGs, while only 1 SNP rs113125564 at *ZC3H4* was selected when SNP data were analyzed. Epigenetics accounted for 17.4% of the variation in perceived facial age, while genetics captured only 1.1%.

**Table 3:** Variance explained and prediction accuracy calculated from best-performing LASSO models based on large-scale methylation and genetic data, evaluated using ten-fold cross-validation in a total set of samples

Predictor type	*N* of predictors	R^2^	rho	MAE
Wrinkle area residuals*				
Methylation	2	0.011	0.100	0.006
Genetic	129	0.395	0.779	0.0048
Methylation + genetic	221	0.590	0.892	0.004
Perceived facial age acceleration*				
Methylation	50	0.174	0.664	3.475
Genetic	1	0.011	0.195	3.811
Methylation + genetic	73	0.262	0.786	3.281

*Residuals from regressing the relevant skin aging phenotypes on age and sex were analyzed as the outcome variable/phenotype

Mean absolute error (MAE) between predicted and measured/observed phenotypes

Spearman’s rho was calculated to assess the correlation between predicted and measured/observed phenotypes

**Fig. 4:**
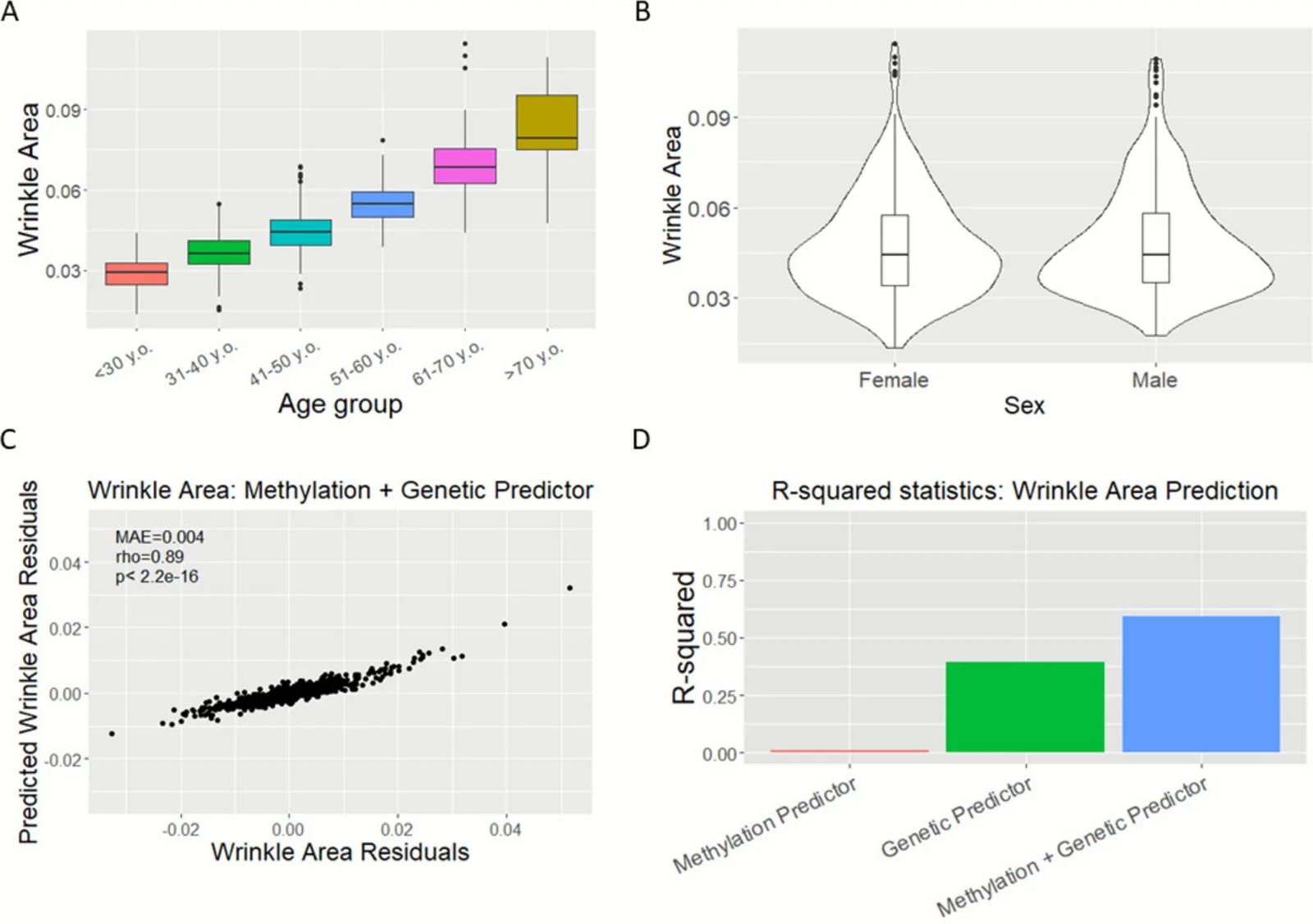
Predictive analysis for wrinkle area using LASSO. **A** Wrinkle area across different age groups (*p* < 2.2 × 10^–16^) in the studied population (*N* = 735, mean wrinkle area = 0.048 ± 0.018, median = 0.044). **B** Violin plot showing sex-related differences in wrinkle area (*p* = 0.566). **C** Predicted vs. measured wrinkle area using large-scale methylation and genetic data, derived from best-performing LASSO model based on tenfold cross-validation; for predictive analyses, the residuals of wrinkle area, termed wrinkle area residuals (i.e., values obtained by regressing wrinkle area on chronological age and sex), served as the outcome variable. **D** R^2^ statistics from LASSO analysis showing the additive effect of methylation and genetic data, accounting for a greater proportion of variance explained compared to using methylation or genetic data alone

**Fig. 5:**
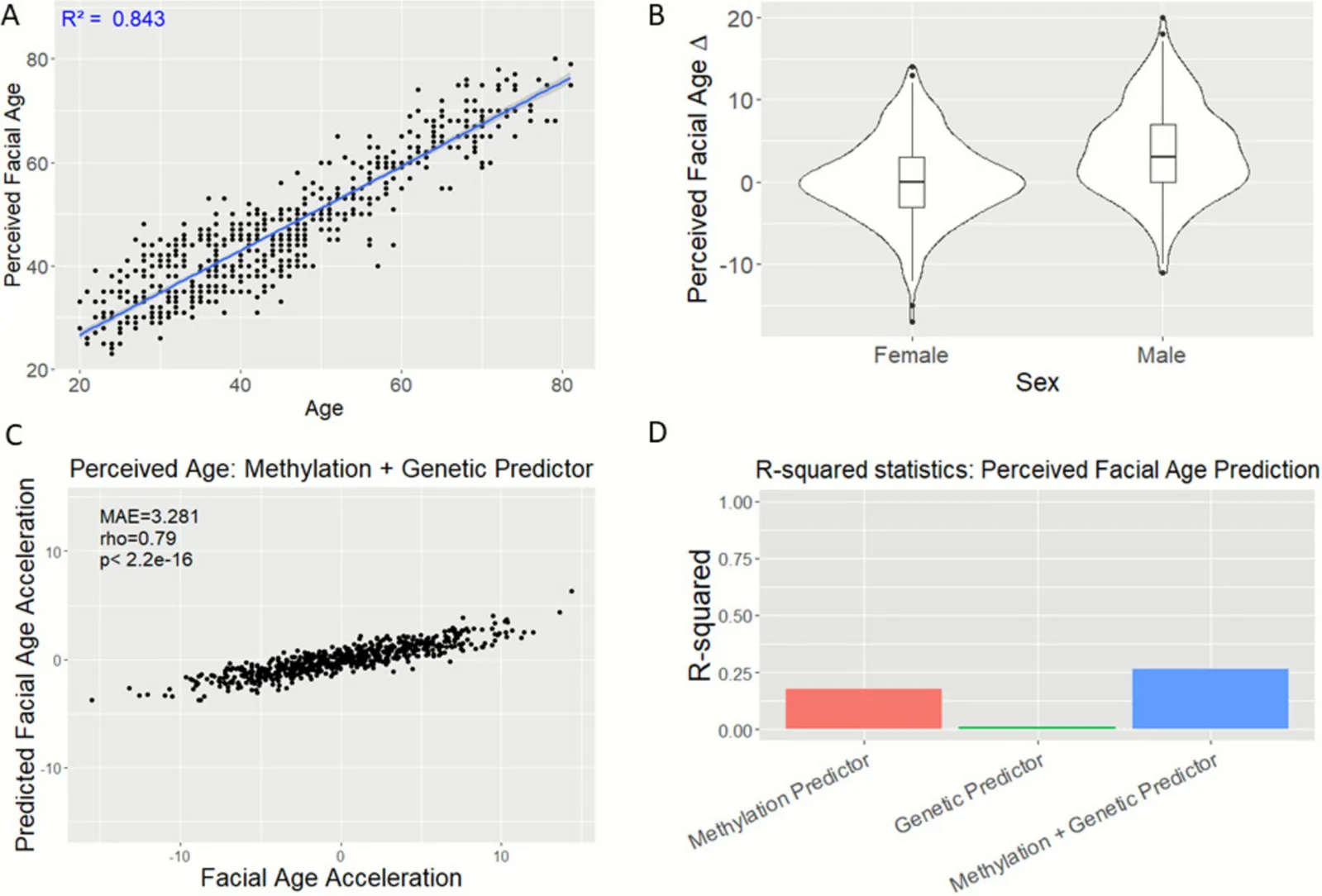
Predictive analysis of perceived facial age using LASSO. **A** Linear plot comparing perceived age with chronological age in the studied population (*N* = 649). **B** Violin plot illustrating sex differences in perceived facial age (*p* = 5.3 × 10^–15^); perceived facial age delta (Perceived Facial Age Δ) refers to the simple difference between perceived facial age and chronological age. **C** Predicted versus observed perceived age based on large-scale methylation and genetic data from the top-performing LASSO model, evaluated using tenfold cross-validation; for predictive analyses, the residuals of perceived age, termed perceived facial age acceleration (i.e., values obtained by regressing perceived age on chronological age and sex), served as the outcome variable. **D** R^2^ statistics from the LASSO analysis showing the combined effect of methylation and genetic data, which account for a larger proportion of the explained variance compared to using either methylation or genetic data alone

In the next step, feature selection with LASSO was rerun using combined methylation and genetic data as input, resulting in better-fitting models (Table 3). The model for predicting wrinkle area incorporated 126 SNPs and 95 CpG markers, with the strongest CpG predictor being cg07283994 at *OCA2* and the strongest SNP predictor being rs73943403 at RP11-78F17.1 (Fig. 4). In contrast, the model for predicting perceived facial age was based on 37 SNPs and 36 CpGs, with the most influential predictors being cg17324588 and cg04570604 at *EID2B* (Fig. 5). From SNP variants, the strongest impact on prediction had rs113125564 at *ZC3H4*. The final models, which incorporate both epigenetic and genetic data, predicted wrinkle area residuals with a mean absolute error (MAE) of 0.004 and perceived facial age acceleration with an MAE of 3.281. The percentage of explained variance was R^2^ = 59.0% for wrinkle area and 26.2% for perceived facial age, respectively. The coefficients extracted from the models with the lowest mean cross-validation error estimate are provided in Supplementary Tables 13–18.

## Discussion

### The skin model of aging

Studying and intervening with biological age and lifespan genes is promising to reduce the prevalence of chronic disease and healthcare burden, and slow down phenotypic signs of aging, offering avenues for many potential applications [50]. However, aging is a highly complex and heterogeneous process, making it difficult to capture with a single diagnostic tool [51]. Skin and its changes over time are a visible manifestation of aging and provide an exceptional model for studying aging processes. Here, we comprehensively analyzed facial skin aging traits, which are characterized by significant diversity, and provided additional evidence that age-correlated facial phenotypes are driven by both genetics, epigenetics, and environmental stressors [52].

### Facial appearance and lifestyle choices

Previous studies have provided strong evidence that excessive exposure to UV radiation induces various structural and molecular changes in the skin [53]. In our study, solarium use or frequent tanning was significantly associated with an increased perceived facial age and deeper nasolabial folds. Interestingly, we also found that sedentary occupations were associated with a younger perceived facial age, less photoaging, less eyelid drooping, and less telangiectasia compared to manual occupations, which are typically associated with greater sun exposure and UV radiation. However, physical activity was associated with improved skin condition in our study, as measured by several skin aging traits. Notably, regular moderate activity has been shown to trigger the expression of antioxidant enzymes and protect the skin from damage caused by reactive oxygen species (ROS) involved in oxidative stress [54]. We also found that practicing yoga showed an association with decreased crow’s feet wrinkles and subjective aging rate. The beneficial effect of yoga on epigenetic aging was described in our previous study [42].

The next factor that had a statistically significant impact on a number of measures that describe skin aging in the population studied was stress. Previous studies have suggested that psychological stress activates mast cells in the skin, and these activated mast cells can produce stress hormones and inflammatory factors [55]. Furthermore, smoking was found to be significantly associated with facial wrinkles. These findings align with previous research that has provided in vitro and in vivo evidence suggesting that smoking tobacco accelerates skin aging [56, 57].

### Blood-based DNA methylation signature reflects facial skin aging

The role of epigenetics in skin functions, homeostasis, and aging is documented in the literature [26, 58–60]. Here, significant hypermethylated and hypomethylated DMPs were detected for wrinkle area, wrinkle length, photonumeric full-face wrinkle score, lip wrinkles, and pigmentation spots. Among the mapped *loci*, the ectodysplasin A receptor (*EDAR*) gene encodes a member of the tumor necrosis factor (TNF) receptor family with a well-described role in the development of ectodermal tissues such as skin and skin appendages [61]. *EDAR* rs3827760 shows robust association with physical appearance traits, including head hair shape [62–65], head hair thickness or density [66, 67], baldness [68], eyebrow thickness [64, 65], beard thickness [65], hairiness [67], unibrow [68], and facial and ear morphology [69], and is therefore a powerful genetic predictor [63, 67]. Genetic variation at *EDAR* was also associated with skin changes and diseases [61, 70]. To the best of our knowledge, this is the first study to report an association between DNA methylation at *EDAR* and facial wrinkling (hypomethylation at cg02925966 is linked to an increased wrinkle area). Importantly, the *EDAR* pathway enhances the activity of NF-κB transcription factors, which contribute to wound healing through their anti-inflammatory and antioxidant properties [71], while impaired wound healing is recognized as one of the hallmarks of aged skin. Furthermore, it has been recently shown that intervening with pathways improved fine wrinkles under the eyes [72].

Also, in this study, differentially hypomethylated CpGs were found in the *NRG1* gene which encodes a member of the epidermal growth factor family, a membrane glycoprotein responsible for cellular signaling and tissue regeneration. Neuregulin 1 glycoprotein plays an important role in several key processes related to skin health and aging, again including wound healing and the growth and migration of keratinocytes and fibroblasts, which are essential for skin renewal [73, 74]. This protein plays a crucial role as a direct ligand for the members of tyrosine kinase receptors of the ERBB signaling pathway which is reported to be involved in regulating skin inflammation after ultraviolet irradiation [75].

### Generations of epigenetic clocks and their link to facial skin aging

There is a clear gap in research investigating the link between epigenetic age and facial appearance or skin condition. One of the few studies found no association between facial age and epigenetic aging, but that study included a smaller selection of epigenetic clocks and was only designed to address perceived facial aging [17]. In contrast, our study shows that facial skin aging parameters are significantly associated with increased epigenetic aging measured in blood. The GrimAge, FitAge, and PoAm/PACE clocks showed the most robust associations, with the first two belonging to the second generation and the latter to the third generation of epigenetic clocks which are thought to reliably capture biological age. All three clocks were associated with how old we look to others (perceived facial age) and how old we feel (subjective rate of aging). GrimAge and PACE were also associated with telangiectasis, and GrimAge was significantly associated with facial wrinkle area and photoaging. The literature emphasizes the superiority of second-generation epigenetic clocks over first-generation clocks for clinical applications, and the particular importance of third-generation clocks for interventional studies [7, 10, 40]. To our knowledge, this is the first study to associate epigenetic age acceleration measured by the GrimAge and FitAge clocks with facial skin aging traits.

Our analyses highlight mechanisms and epigenetic changes that correlate with aging-related facial phenotypes in blood samples. Although this may be considered a limitation, recent studies have shown that facial aging may reflect systemic aging observed at multiple organ levels [76]. Furthermore, the vast majority of epigenetic clocks have been developed in blood samples, and previous studies have also observed a correlation between the epigenetic pace of aging and facial appearance tested in blood samples [40].

### Genetic variants and facial skin aging

Previous GWAS studies have contributed to the understanding of the genetic factors underlying skin aging traits. For instance, skin damage was found to be associated with rs185146 at *SLC45A2*, rs12203592 at *IRF4*, and rs4268748 at *MC1R*, implicating pigmentation genes in the skin aging process [77]. Another recent study reported 4 novel SNPs associated with facial skin wrinkles and 6 SNPs for pigmentation spots [78]. Importantly, specific genes associated with different features of skin aging are determined only to a very limited extent. Interestingly, one of the strongest predictors of facial wrinkling in our study was the methylation of the *OCA2* gene, which, along with the previously mentioned genes, is among the key pigmentation genes [79].

Our study revealed novel genome-wide significant associations. The rs73943403 (A>G) located on chromosome 18 and mapped to the RP11-78F17.1 gene showed a significant association with wrinkle area. RP11-78F17.1 (lnc-ADCYAP1) encodes a long non-coding RNA with *ADCYAP1* as a potential candidate gene under regulation. In turn, the protein product of *ADCYAP1*, the neuropeptide PACAP, is responsible for stimulating adenylate cyclase activity and increasing cyclic adenosine monophosphate (cAMP) levels. It acts as a master regulator of the cellular stress response [80], exhibits anti-apoptotic and anti-inflammatory properties, and has a documented role in cellular and tissue aging, providing protection against a variety of damaging stimuli [81, 82]. Interestingly, genetic variants in *ADCYAP1* have been associated with extreme parental longevity [83]. *ADCYAP1* is also involved in the control of glucose homeostasis and has been associated with BMI and obesity-related traits [84, 85], while skin alterations related to skin collagen structure, wound healing, and mechanical friction have been widely reported in obese individuals [86, 87].

The intronic SNP, rs113125564 (T>C) located on chromosome 19 mapped to the *ZC3H4* gene, showed a genome-wide significant association with perceived facial age. *ZC3H4* encodes a zinc finger protein involved in extragenic and long non-coding RNA transcription. Through gene expression regulation, *ZC3H4* has been suggested to impact metabolic pathways related to obesity, potentially through inflammatory pathways [88], while low-grade inflammation is often present in obesity-related traits [89]. Furthermore, multiple studies have established a link between genetic variants at *ZC3H4* and obesity or eating behaviors [90, 91]*.*

Our finding linking RP11-78F17.1 and *ZC3H4* to facial aging traits may therefore support the role of stress response and adiposity in skin aging, while also highlighting the importance of gene regulatory mechanisms in this process.

### Predictive analysis

The literature mostly describes predictive models related to perceived facial age, with only some of them relying on methylation data analysis [17, 18], while others are based on digital facial image analysis [92, 93]. In 2018, Marioni et al. used top CpGs from an epigenome-wide association study in blood samples and developed an epigenetic predictor of facial age that correlated with phenotypic facial age at *r* = 0.66 and predicted an increased risk of mortality [17]. In turn, in 2024, Bienkowska et al. [18] developed the first epigenetic clock tailored to skin tissue [18] that predicted visual facial age and facial age progression using DNA methylation data and was shown to be useful for evaluating specific skin interventions [94].

In this study, we evaluated the potential of DNA methylation and SNP data to predict two facial skin aging phenotypes, including wrinkle area and perceived facial age. While wrinkle area is based on precise digital quantification of wrinkles and could be useful and easily applied in facial reconstructions in forensic anthropology, perceived facial age is thought to provide a more holistic view, capturing more than one aspect of skin aging [18]. The analyses performed showed that methylation data and SNPs explained a significant portion of the variability in facial skin aging traits, highlighting their additive value. These analyses provide a foundation for future studies, which should focus on careful selection of CpGs and SNPs, the training process, and model validation on independent sample sets.

### Limitations of the study

Although the findings of this study enhance our understanding of the epigenetic and genetic contributions to skin aging, certain limitations must be acknowledged. The relatively small sample size constitutes a primary limitation, precluding replication of the results in an independent cohort. The absence of an independent group for predictive analysis constitutes an additional constraint; the initial model developed in this work requires evaluation in future studies using independent cohorts, ideally drawn from different populations. Furthermore, while perceived age was assessed by a professional dermatologist, the lack of additional raters presents another limitation, given that the inherently subjective nature of perceived age assessment may reduce reproducibility across studies. Finally, although blood-derived DNA methylation can serve as a surrogate for skin aging phenotypes, future validation in skin tissue methylation data would strengthen the translational relevance of our findings. Potential tissue-specific methylation differences and their impact on result interpretation warrant further investigation.

## Conclusions

In response to the human desire to look younger and live longer while staying healthy, a better understanding of the genes and molecular pathways involved in skin aging is vital. The study demonstrated that age-related facial skin phenotypes are influenced by multiple stressors, including socioeconomic, lifestyle, and environmental factors, such as education, socioeconomic status, physical activity, yoga practice, stress, BMI, and dose-dependent smoking. We have shown that facial skin aging significantly correlates with epigenetic aging measured in blood, and the tools that most robustly capture the variability observed in facial skin aging phenotypes are GrimAge, FitAge, and PACE. This highlights the relevance of second- and third-generation epigenetic clocks for future skin aging research and targeted interventions. We confirmed that aging skin exhibits a unique epigenetic pattern reflected in blood, and discovered new differentially methylated genes, including *EDAR* and *NRG1*, known to be engaged in wound healing and facial nerve injury repair. The discovery of the association between DNA methylation in the *EDAR* gene and skin aging is groundbreaking, as SNP variation in this gene has been repeatedly associated with various phenotypes. Given that DNA methylation reflects environmental exposure and is reversible, *EDAR* represents an ideal target for future skin interventions.

## Supplementary Information

Below is the link to the electronic supplementary material.

ESM 1 (PDF 1.00 MB)

ESM 2 (XLSX 208 KB)

## Data Availability

A portion of the data supporting the findings of this study is presented in the Supplementary Tables. Additional data are available from the authors upon request, but restrictions apply due to the project policy.
