# Supplementary material for: Facial skin aging: an integrative analysis of genetics, epigenetics, and lifestyle factors
Source: GeroScience. 2025 Dec 24;48(6):7627–44. doi: 10.1007/s11357-025-02067-w (PMC13638814; doi:10.1007/s11357-025-02067-w)
Supplement: Supplementary file 1 [file 11357_2025_2067_MOESM1_ESM.pdf]

Supplementary Methods

Skin aging phenotyping methods

Multiple phenotypes were generated in the project. Photonumerical classification and semi-automated computational quantitative methods were used to define facial skin aging phenotypes. For all participants, 2D facial images (three angles including full face, right, and left profiles) and high-resolution 3D facial scans were obtained under optimized conditions with a standardized viewpoint, appropriate lighting, without makeup or jewelry using the VECTRA® H1 imaging system (Canfield Scientific, Inc.). Preprocessing of 3D scans and conversion of 3D to 2D photographs was performed using VECTRA® H1 software version 6.2. Semi-automated facial wrinkle detection and quantification were done using MATLAB software (MATLAB. (2018). 9.7.0.1190202 (R2019b). Natick, Massachusetts: The MathWorks Inc.). The grayscale images were preprocessed and smoothed by applying the Gaussian filter [1] and masked to isolate the face skin area of interest, excluding eyes, eyebrows, nose, and mouth areas. Wrinkles were detected using the Canny Edge Detector algorithm [2]. The detected wrinkles were quantified by calculating facial wrinkle area (pixels detected as wrinkles as a percentage of the total skin area) and wrinkle length (length of areas detected as wrinkles normalized by the square root of the total skin area) [3].

Assessment of other facial aging traits was also conducted either by a board-certified dermatologist or by the participants themselves, and included the following: i) **perceived facial age** estimation, determined by visual assessment of the participant's facial appearance without prior knowledge of chronological age [4]; perceived age was assessed by a professional dermatologist involved in the project, who performed the evaluation based on 3D facial scans and 2D photographs; in the first stage, following an initial analysis, the dermatologist estimated the age within a ±5-year range; during the second assessment, a more precise perceived age was assigned based on the person’s facial appearance; ii) **subjective aging rate**, determined by the participants themselves based on how old they felt (0 - younger than real age, 1 - same as real

age, 2 - older than real age); iii) **the Glogau classification of photoaging** to categorize skin types from I to IV, indicating respectively: no wrinkles, wrinkles in motion, wrinkles at rest, and wrinkles only [5]; iv) the photonumeric/binary score of facial skin aging to assess **the presence of wrinkles** in different areas of the face, including the full face, forehead, eye area (crow's feet wrinkles), and lip wrinkles. Measurements were classified as either deep or small. The presence of eyelid drooping, nasolabial folds, pigmentation spots, telangiectasia, facial height and lip height were also recorded. Additionally, the spectrophotometric measurement of skin color using the Fitzpatrick skin typing scale was determined (**Supplementary Table 3).**

DNA methylation quantification

DNA from blood for all samples collected in the study (N=735) was extracted using an automated method and the Maxwell RSC Blood DNA Kit (Promega Corporation), followed by determination of purity using NanoDrop (Thermo Scientific, MA, USA) and concentration using the Qubit dsDNA HS Assay Kit (Thermo Fisher Scientific). The amount of 500 ng DNA per sample was subjected to bisulfite conversion using the EZ-96 DNA Methylation Kit (Zymo Research Corp., CA, USA) according to the manufacturer's instructions for Infinium assays. DNA samples were randomized using the web-based application RANDOMIZE [6], and the methylation profile for all samples was obtained using the Illumina Infinium Methylation EPIC microarray (Illumina, San Diego, CA, USA). Primary quality control of the generated DNA methylation data was performed using the GenomeStudio software and the Methylation Module v1.8 (Illumina Inc, 2008) [7]. Further sample and probe-based quality control was performed in R version 4.2 using the minfi [8] and limma [9] R packages. A p-value threshold of 0.05 and 0.01 was used for probe and sample call rates, respectively. Two types of normalization, quantile normalization, were applied for the downstream epigenetic age estimation and epigenome-wide association (EWAS) analyses, respectively. The methylation level was

calculated as the β value (the ratio of the methylated signal to the total signal per probe, with an added constant in the denominator ranging from 0 and 1). In addition, for EWAS further probe filtering steps were performed to exclude the list of probes if they were I) mapped to X and Y chromosomes (except for hair loss), II) reported to overlap with SNPs, III) cross-reactive mapped to multiple genomic locations [10], IV) problematic probes reported by Illumina.

DMPs significantly associated (FDR $p < 0.05$) with specific phenotypes were identified through a series of EWAS analyses and using the limma package [9]. Linear models were fitted using derived methylation M-values, the log2 ratio of the intensities obtained from methylated probes to unmethylated probes. In fitted model 1, the results of the EWAS analysis were adjusted for age, sex, and cell components. Model 2 was additionally adjusted for environmental factors.

Profiling of epigenetic clocks and associations with facial skin aging

Epigenetic age was estimated for all samples studied using the methylCIPHER R package for several DNAm-based biomarkers, including DNAm age Horvath2013, DNAm age Hannum, DNAm age Skin&Blood, DNAm PhenoAge, DNAm GrimAge, and DNAm FitAge [11–17]. The methylation level, represented as the β value, was used as the input. The corresponding epigenetic age acceleration (EAA) measures were calculated as the residuals of regressing DNAm age on chronological age. EAA measures included the intrinsic epigenetic age acceleration (IEAA) calculated by regressing the Horvath2013 clock on chronological age, extrinsic epigenetic age acceleration (EEAA) estimated by regressing the Hannum clock on chronological age, DNAm PhenoAge acceleration (PhenoAgeAccel), DNAm GrimAge acceleration (GrimAgeAccel), DNAm FitAge acceleration (FitAgeAccel). In addition, DNAm-based pace of aging (PACE and PoAm) and epigenetic Mortality Risk Score (Zhang MRS) were measured [18–20]. The Steve Horvath & Clock Foundation online algorithm

(https://dnamage.clockfoundation.org/) was used to estimate the methylation-based blood cell components [11,21]. For missing CpGs included in different clocks, we followed the imputation procedure described in the Horvath online calculator. Specifically, k-nearest neighbor (kNN) imputation with k = 10 was applied to fill in missing values within each clock. For CpGs missing across all samples (i.e., probes not covered by the Illumina EPIC v1 array), we used the Horvath "gold-standard" imputation approach, replacing missing values with the mean values provided in the datMiniAnnotation3 reference list. For calculating DunedinPACE, 173 probes were used, and any missing values were replaced with the cohort-specific mean, as described in the official DunedinPACE GitHub documentation [18]. A default threshold of 0.7 was applied.

The EAA measures were next tested for association with different skin and hair phenotypes. Model 1 was adjusted for sex and age, and model 2 was additionally adjusted for smoking status, stress level, skin color, frequency of tanning or solarium use, and blood cell proportions.

Genome-wide association analyses

The Global Screening Array (GSA) from Illumina (San Diego, CA) was used for sample genotyping. Quality control prior to imputation was performed using PLINK1.9 [40] Samples or SNPs with more than 5% missing data were excluded, and SNPs with significant deviation from Hardy-Weinberg equilibrium were filtered based on $p < 1.0 \times 10^{-6}$. The minor allele frequency (MAF) threshold was set at <0.01. Only biallelic SNPs on autosomal chromosomes mapped to a unique genomic location were retained for further analysis. Principal component analysis (PCA) was performed on the pruned variants to detect population stratification or genetic outliers. SNPs were then imputed using Beagle version 5.4 [41]. The final number of 719 samples and 477,827 SNPs were used for genome-wide association analyses (GWAS)

performed using linear regression for testing genetic association with continuous skin aging phenotypes and logistic regression for binary skin aging traits under an additive genetic model. Multivariate regression models were adjusted for age, sex, BMI, skin color, smoking status, DNA methylation pack-years, stress level, solarium exposure, tanning frequency, and a p-value threshold of $<5 \times 10^{-8}$ was considered statistically significant.

References

[1] R. González, R. Woods, S.L. Eddins, Digital image processing using MATLAB, (2006).

[2] Y.M. Luo, R. Duraiswami, Canny edge detection on NVIDIA CUDA, 2008 IEEE Computer Society Conference on Computer Vision and Pattern Recognition Workshops (2008). https://doi.org/10.1109/CVPRW.2008.4563088.

[3] M.A. Hamer, L.C. Jacobs, J.S. Lall, A. Wollstein, L.M. Hollestein, A.R. Rae, K.W. Gossage, A. Hofman, F. Liu, M. Kayser, T. Nijsten, D.A. Gunn, Validation of image analysis techniques to measure skin aging features from facial photographs, Skin Research and Technology 21 (2015) 392–402. https://doi.org/10.1111/SRT.12205.

[4] M. Coma, R. Valls, J.M. Mas, A. Pujol, M.A. Herranz, V. Alonso, J. Naval, Methods for diagnosing perceived age on the basis of an ensemble of phenotypic features, Clin Cosmet Investig Dermatol 7 (2014) 133–137. https://doi.org/10.2147/CCID.S52257.

[5] R.G. Glogau, Aesthetic and anatomic analysis of the aging skin., Semin Cutan Med Surg 15 (1996) 134–138. https://doi.org/10.1016/S1085-5629(96)80003-4.

[6] A.H. Wani, J. Dahrendorff, M. Uddin, RANDOMIZE: A Web Server for Data Randomization, (n.d.). https://doi.org/10.1101/2020.04.02.013656.

[7] Illumina, GenomeStudio Methylation Module v1.8 User Guide (11319130), (2010).

[8] J.P. Fortin, T.J. Triche, K.D. Hansen, Preprocessing, normalization and integration of the Illumina HumanMethylationEPIC array with minfi, Bioinformatics 33 (2017) 558. https://doi.org/10.1093/BIOINFORMATICS/BTW691.

[9] M.E. Ritchie, B. Phipson, D. Wu, Y. Hu, C.W. Law, W. Shi, G.K. Smyth, limma powers differential expression analyses for RNA-sequencing and microarray studies, Nucleic Acids Res 43 (2015) e47. https://doi.org/10.1093/NAR/GKV007.

[10] Y.-A. Chen, M. Lemire, S. Choufani, D.T. Butcher, D. Grafodatskaya, B.W. Zanke, S. Gallinger, T.J. Hudson, R. Weksberg, Discovery of cross-reactive probes and polymorphic CpGs in the Illumina Infinium HumanMethylation450 microarray, Epigenetics 203 Epigenetics 8 (2013) 203–209. https://doi.org/10.4161/epi.23470.

[11] S. Horvath, DNA methylation age of human tissues and cell types, Genome Biol 14 (2013). https://doi.org/10.1186/gb-2013-14-10-r115.

[12] A.T. Lu, A. Quach, J.G. Wilson, A.P. Reiner, A. Aviv, K. Raj, L. Hou, A.A. Baccarelli, Y. Li, J.D. Stewart, E.A. Whitsel, T.L. Assimes, L. Ferrucci, S. Horvath, DNA methylation GrimAge strongly predicts lifespan and healthspan, Aging (Albany NY) 11 (2019) 303. https://doi.org/10.18632/AGING.101684.

[13] M.E. Levine, A.T. Lu, A. Quach, B.H. Chen, T.L. Assimes, S. Bandinelli, L. Hou, A.A. Baccarelli, J.D. Stewart, Y. Li, E.A. Whitsel, J.G. Wilson, A.P. Reiner1, A. Aviv1, K. Lohman, Y. Liu, L. Ferrucci, S. Horvath, An epigenetic biomarker of aging for lifespan and healthspan, Aging 10 (2018) 573–591. https://doi.org/10.18632/AGING.101414.

[14] K.M. McGreevy, Z. Radak, F. Torma, M. Jokai, A.T. Lu, D.W. Belsky, A. Binder, R.E. Marioni, L. Ferrucci, E. Pośpiech, W. Branicki, A. Ossowski, A. Sitek, M. Spólnicka, L.M. Raffield, A.P. Reiner, S. Cox, M. Kobor, D.L. Corcoran, S. Horvath, DNAmFitAge: biological age indicator incorporating physical fitness, Aging 15 (2023). https://doi.org/10.18632/AGING.204538.

[15] S. Horvath, J. Oshima, G.M. Martin, A.T. Lu, A. Quach, H. Cohen, S. Felton, M. Matsuyama, D. Lowe, S. Kabacik, J.G. Wilson, A.P. Reiner, A. Maierhofer, J. Flunkert, A. Aviv, L. Hou, A.A. Baccarelli, Y. Li, J.D. Stewart, E.A. Whitsel, L. Ferrucci, S. Matsuyama, K. Raj, Epigenetic clock for skin and blood cells applied to Hutchinson Gilford Progeria Syndrome and ex vivo studies, Aging 10 (2018) 1758–1775. https://doi.org/10.18632/AGING.101508.

[16] G. Hannum, J. Guinney, L. Zhao, L. Zhang, G. Hughes, S.V. Sadda, B. Klotzle, M. Bibikova, J.B. Fan, Y. Gao, R. Deconde, M. Chen, I. Rajapakse, S. Friend, T. Ideker, K. Zhang, Genome-wide Methylation Profiles Reveal Quantitative Views of Human Aging Rates, Mol Cell 49 (2013) 359–367. https://doi.org/10.1016/j.molcel.2012.10.016.

[17] A.T. Lu, A. Seeboth, P.C. Tsai, D. Sun, A. Quach, A.P. Reiner, C. Kooperberg, L. Ferrucci, L. Hou, A.A. Baccarelli, Y. Li, S.E. Harris, J. Corley, A. Taylor, I.J. Deary, J.D. Stewart, E.A. Whitsel, T.L. Assimes, W. Chen, S. Li, M. Mangino, J.T. Bell, J.G. Wilson, A. Aviv, R.E. Marioni, K. Raj, S. Horvath, DNA methylation-based estimator of telomere length, Aging 11 (2019) 5895–5923. https://doi.org/10.18632/AGING.102173.

[18] D.W. Belsky, A. Caspi, D.L. Corcoran, K. Sugden, R. Poulton, L. Arseneault, A. Baccarelli, K. Chamarti, X. Gao, E. Hannon, H.L. Harrington, R. Houts, M. Kothari, D. Kwon, J. Mill, J. Schwartz, P. Vokonas, C. Wang, B. Williams, T.E. Moffitt, DunedinPACE, a DNA methylation biomarker of the pace of aging, Elife 11 (2022). https://doi.org/10.7554/ELIFE.73420.

[19] D.W. Belsky, A. Caspi, L. Arseneault, A. Baccarelli, D. Corcoran, X. Gao, E. Hannon, H.L. Harrington, L.J.H. Rasmussen, R. Houts, K. Huffman, W.E. Kraus, D. Kwon, J. Mill, C.F. Pieper, J. Prinz, R. Poulton, J. Schwartz, K. Sugden, P. Vokonas, B.S. Williams, T.E. Moffitt, Quantification of the pace of biological aging in humans through a blood test, the DunedinPoAm DNA methylation algorithm, Elife 9 (2020) 1–56. https://doi.org/10.7554/ELIFE.54870.

[20] Y. Zhang, R. Wilson, J. Heiss, L.P. Breitling, K.U. Saum, B. Schöttker, B. Holleczek, M. Waldenberger, A. Peters, H. Brenner, DNA methylation signatures in peripheral blood strongly predict all-cause mortality, Nat Commun 8 (2017). https://doi.org/10.1038/NCOMMS14617.

[21] E.A. Houseman, W.P. Accomando, D.C. Koestler, B.C. Christensen, C.J. Marsit, H.H. Nelson, J.K. Wiencke, K.T. Kelsey, DNA methylation arrays as surrogate measures of cell mixture distribution, BMC Bioinformatics 13 (2012) 86. https://doi.org/10.1186/1471-2105-13-86.

Supplementary Figures

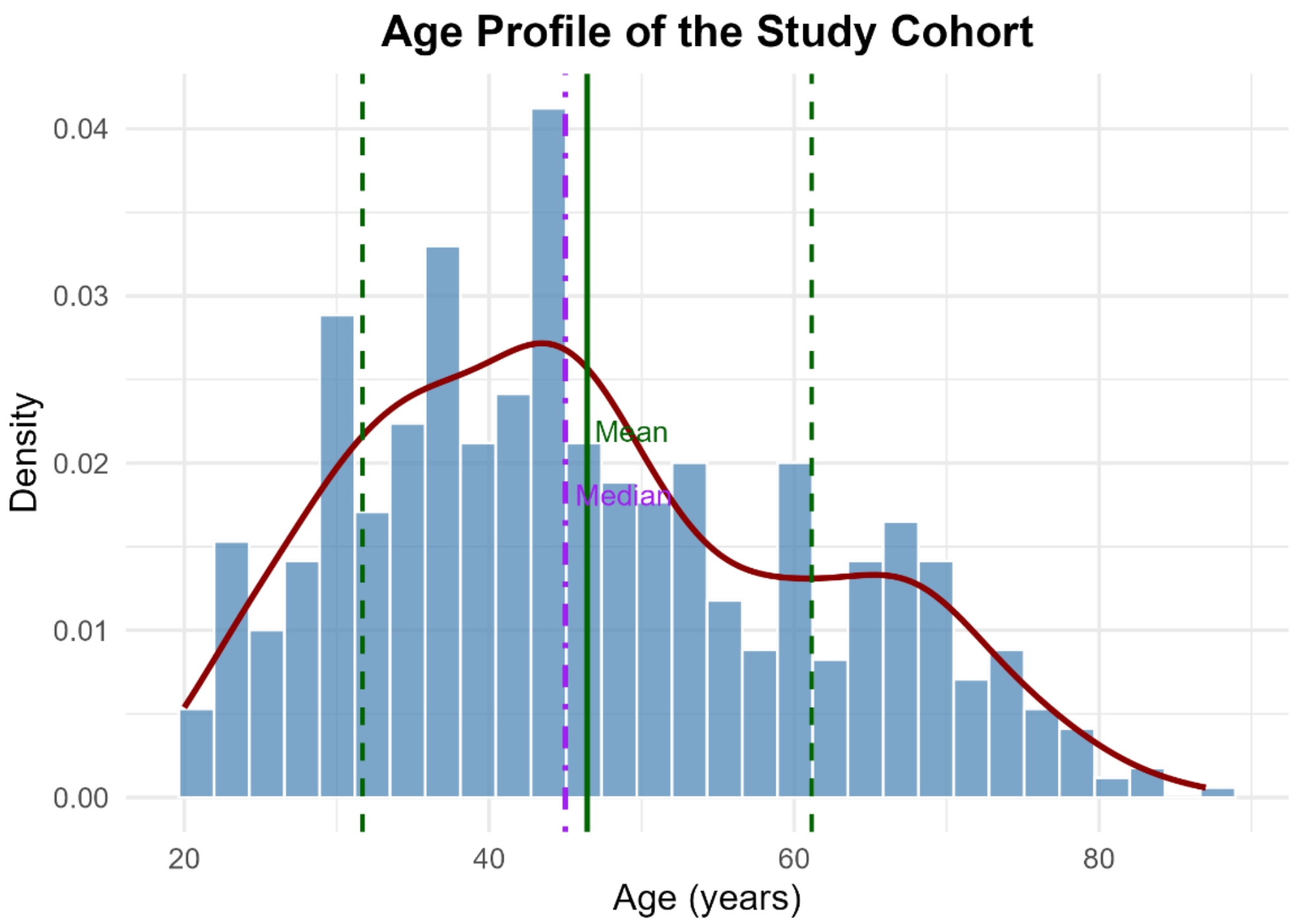

Supplementary Figure 1. Age profile of the studied population (N=735): histogram. Mean ± SD: 46.4 ± 14.7 years; Median: 45 years.

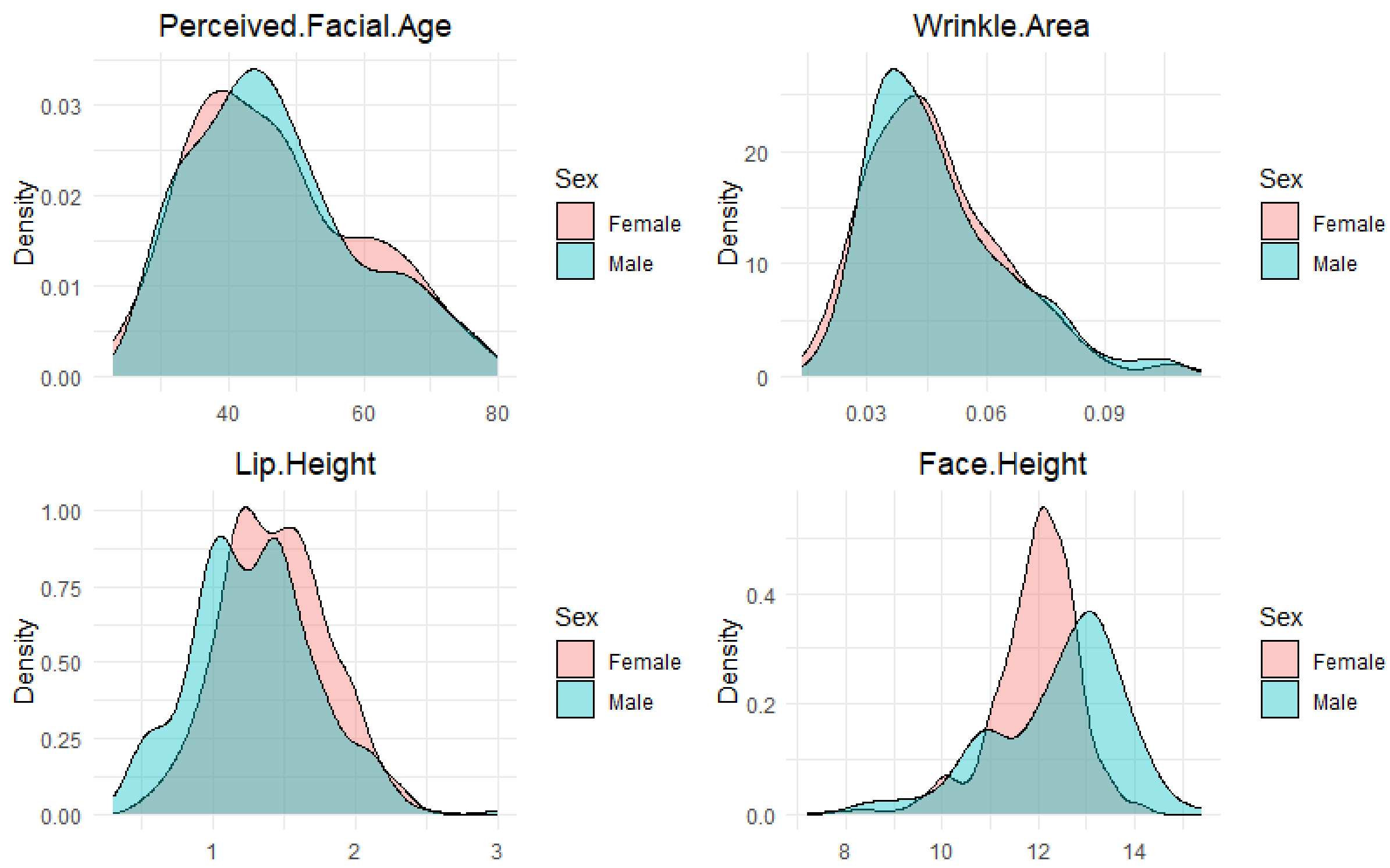

Supplementary Figure 2. Density plots of quantitative skin-aging phenotypes analysed in the study.

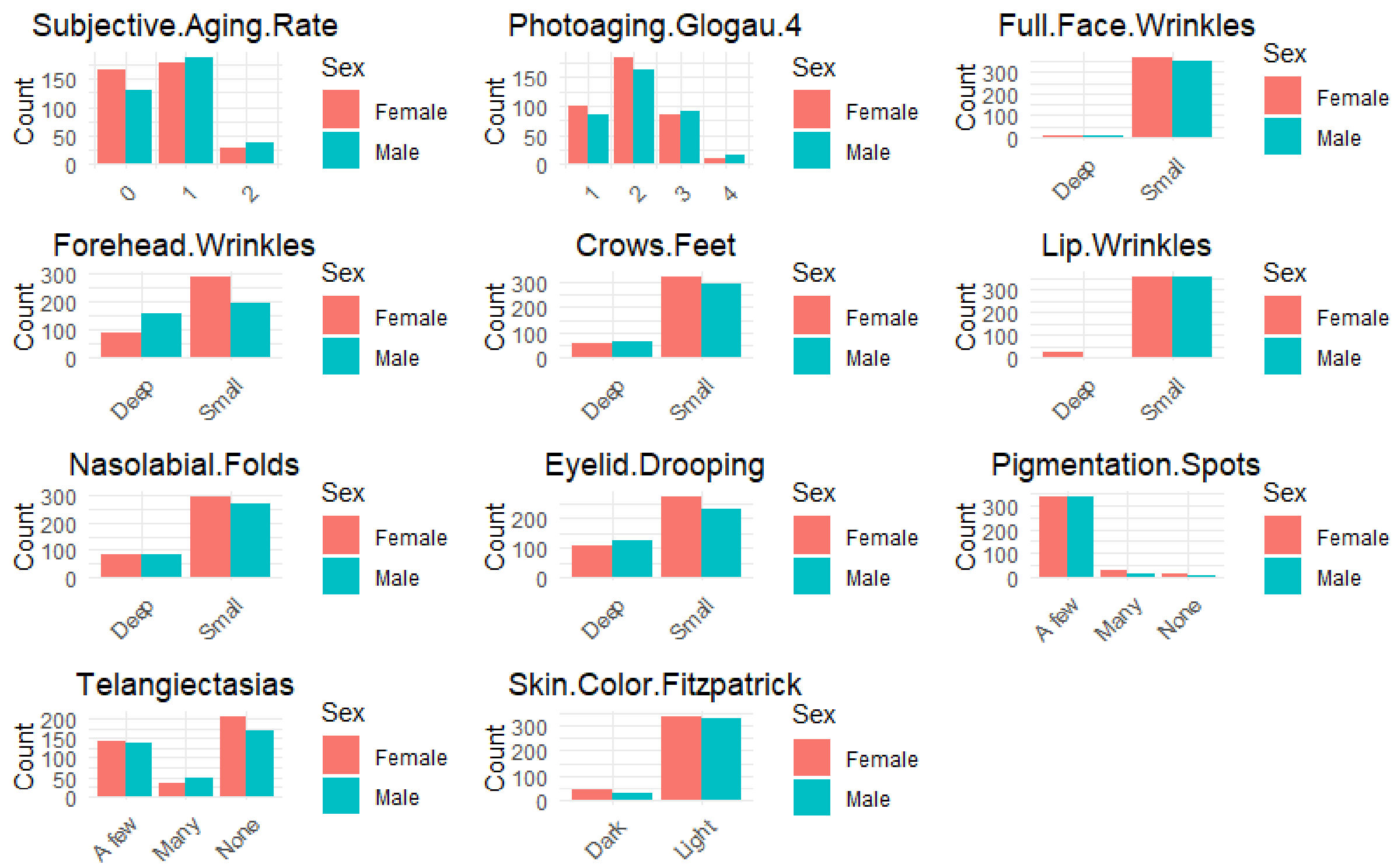

Supplementary Figure 3. Bar plots for categorical skin aging phenotypes analysed in the study.

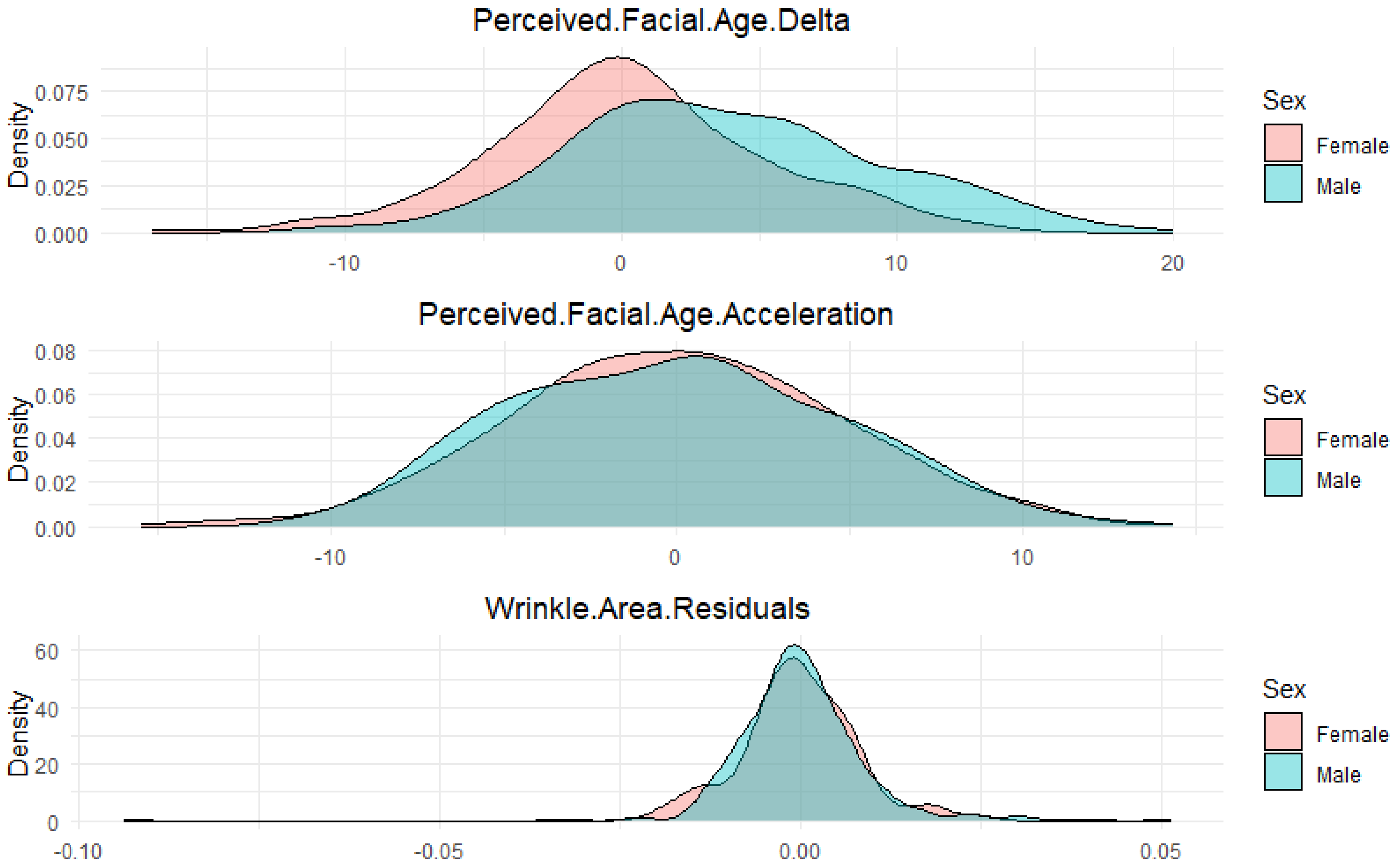

Supplementary Figure 4. Adjusted skin aging phenotype distribution. To remove the effects of confounding factors for the purposes of predictive analysis, instead of using absolute values the phenotypes wrinkle area and perceived facial age were adjusted for the effects of age and/or sex. The parameter perceived facial age delta represents the simple difference between perceived age and chronological age. The parameters perceived facial age acceleration and wrinkle area residuals are calculated as the residuals from regression of perceived facial age and wrinkle area on both chronological age and sex.

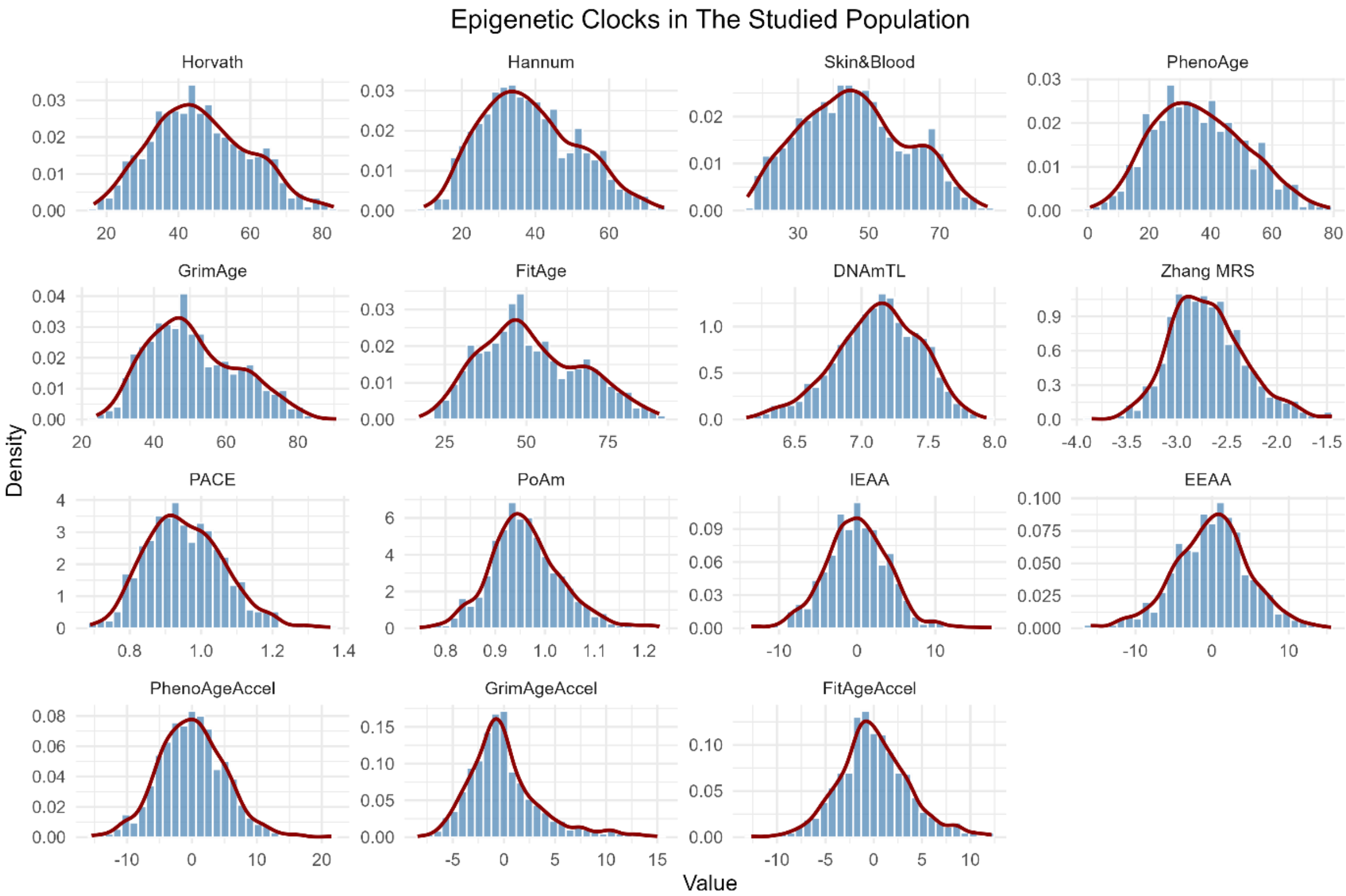

Supplementary Figure 5. Epigenetic clock profiles across the study population (N=735): histogram representation.

Supplementary Tables

Supplementary Tables 5, 6, 7, 8, 10, 13, 14, 15, 16, 17, and 18 are provided in a separate Excel file.

Supplementary Table 1. Demographic information of study participants.

	Female (N=379)	Male (N=356)	All subjects (N=735)
**Age**			
Mean (SD)	47.8 (13.7)	45.0 (15.6)	46.4 (14.7)
**Education**			
University	256 (67.5%)	225 (63.2%)	481 (65.4%)
No University	123 (32.5%)	130 (36.5%)	253 (34.4%)
Missing	0 (0%)	1 (0.3%)	1 (0.1%)
**Socioeconomic status (SES)**			
Low-SES	21 (5.5%)	8 (2.2%)	29 (3.9%)
Medium-SES	284 (74.9%)	249 (69.9%)	533 (72.5%)
High-SES	72 (19.0%)	96 (27.0%)	168 (22.9%)
Missing	2 (0.5%)	3 (0.8%)	5 (0.7%)
**Area of residence**			
Village	64 (16.9%)	59 (16.6%)	123 (16.7%)
City<100K	63 (16.6%)	59 (16.6%)	122 (16.6%)
City>100K	251 (66.2%)	236 (66.3%)	487 (66.3%)
Missing	1 (0.3%)	2 (0.6%)	3 (0.4%)
**Type of job**			
Intern/retired/unemployed	70 (18.5%)	55 (15.4%)	125 (17.0%)
Sedentary mental work (SM)	112 (29.6%)	86 (24.2%)	198 (26.9%)
Long hours sedentary mental work (LSM)	159 (42.0%)	120 (33.7%)	279 (38.0%)
Manual (M)	37 (9.8%)	93 (26.1%)	130 (17.7%)
Missing	1 (0.3%)	2 (0.6%)	3 (0.4%)

Supplementary Table 2. Participants' lifestyle information.

	**Female (N=379)**	**Male (N=356)**	**All subjects (N=735)**
**BMI**			
Mean (SD)	25.5 (4.8)	27.6 (4.1)	26.5 (4.6)
Missing	3 (0.8%)	2 (0.6%)	5 (0.7%)
**Smoking status**			
Never smoker	229 (60.4%)	177 (49.7%)	406 (55.2%)
Former smoker	77 (20.3%)	77 (21.6%)	154 (21.0%)
Current smoker	73 (19.3%)	99 (27.8%)	172 (23.4%)
Missing	0 (0%)	3 (0.8%)	3 (0.4%)
**Alcohol drinking**			
Never	52 (13.7%)	34 (9.6%)	86 (11.7%)
Occasionally	295 (77.8%)	268 (75.3%)	563 (76.6%)
Frequently	31 (8.2%)	51 (14.3%)	82 (11.2%)
Missing	1 (0.3%)	3 (0.8%)	4 (0.5%)
**Physical activity (PA)**			
Inactive	92 (24.3%)	61 (17.1%)	153 (20.8%)
Active	287 (75.7%)	294 (82.6%)	581 (79.0%)
Missing	0 (0%)	1 (0.3%)	1 (0.1%)
**Self-report of stress sensitivity**			
FALSE	359 (94.7%)	274 (77.0%)	633 (86.1%)
TRUE	20 (5.3%)	82 (23.0%)	102 (13.9%)
**Yoga/meditation**			
FALSE	324 (85.5%)	343 (96.3%)	667 (90.7%)
TRUE	55 (14.5%)	13 (3.7%)	68 (9.3%)
**Sleeping hours**			
Mean (SD)	6.7 (1.2)	6.7 (1.1)	6.7 (1.1)
Missing	0 (0%)	3 (0.8%)	3 (0.4%)
**Type of diet**			
Varied	357 (94.2%)	332 (93.3%)	689 (93.7%)

	Female (N=379)	Male (N=356)	All subjects (N=735)
Protein	4 (1.1%)	16 (4.5%)	20 (2.7%)
Vegetarian	17 (4.5%)	6 (1.7%)	23 (3.1%)
Missing	1 (0.3%)	2 (0.6%)	3 (0.4%)
**Sugar intake**			
Low	27 (7.1%)	31 (8.7%)	58 (7.9%)
Medium	275 (72.6%)	274 (77.0%)	549 (74.7%)
High	77 (20.3%)	50 (14.0%)	127 (17.3%)
Missing	0 (0%)	1 (0.3%)	1 (0.1%)
**Frequency of tanning**			
Rarely	207 (54.6%)	208 (58.4%)	415 (56.5%)
Frequently	171 (45.1%)	144 (40.4%)	315 (42.9%)
Missing	1 (0.3%)	4 (1.1%)	5 (0.7%)
**Solarium use**			
Never	222 (58.6%)	287 (80.6%)	509 (69.3%)
Former History	129 (34.0%)	54 (15.2%)	183 (24.9%)
Currently	28 (7.4%)	14 (3.9%)	42 (5.7%)
Missing	0 (0%)	1 (0.3%)	1 (0.1%)
**Cups of coffee**			
None	58 (15.3%)	79 (22.2%)	137 (18.6%)
Small (1-2/day)	245 (64.6%)	164 (46.1%)	409 (55.6%)
Large (≥3/day)	69 (18.2%)	109 (30.6%)	178 (24.2%)
Missing	7 (1.8%)	4 (1.1%)	11 (1.5%)

Supplementary Table 3. Facial skin aging characteristics of the study participants.

		Female (N=379)		Male (N=356)		All subjects (N=735)
**Perceived facial age**						
Mean (SD)		47.1 (12.8)		46.9 (12.3)		47.0 (12.5)
Median (Q1, Q3)		45.0 (37.3, 56.0)		45.0 (38.0, 55.0)		45.0 (38.0, 55.0)
Missing		53 (14.0%)		33 (9.3%)		86 (11.7%)
**Subjective aging rate**						
0 (Younger than real age)		165 (43.5%)		130 (36.5%)		295 (40.1%)
1 (As real age)		179 (47.2%)		186 (52.2%)		365 (49.7%)
2 (Older than real age)		30 (7.9%)		38 (10.7%)		68 (9.3%)
Missing		5 (1.3%)		2 (0.6%)		7 (1.0%)
**Wrinkle area %**						
Mean (SD)		4.8 (1.8)		4.8 (1.8)		4.8 (1.8)
Median (Q1, Q3)		4.5 (3.5, 5.8)		4.4 (3.5, 5.8)		4.4 (3.5, 5.8)
**Wrinkle length**						
Mean (SD)		14870.5 (7746.4)		12963.6 (7517.7)		13946.9 (7690.7)
Median (Q1, Q3)		12829.0 (9318.0, 18579.5)		10465.5 (7788.5, 15908.8)		11543.0 (8385.0, 17661.0)
**Photoaging Glogau 4**						
1		101 (26.6%)		85 (23.9%)		186 (25.3%)
2		182 (48.0%)		164 (46.1%)		346 (47.1%)
3		86 (22.7%)		91 (25.6%)		177 (24.1%)
4		10 (2.6%)		16 (4.5%)		26 (3.5%)
**PhotoagingGlogau 2**						
1		283 (74.7%)		249 (69.9%)		532 (72.4%)
2		96 (25.3%)		107 (30.1%)		203 (27.6%)
**Photonumeric full-face wrinkle score**						
Small		367 (96.8%)		346 (97.2%)		713 (97.0%)
Deep		12 (3.2%)		10 (2.8%)		22 (3.0%)
**Forehead wrinkles**						
Small		291 (76.8%)		198 (55.6%)		489 (66.5%)
Deep		88 (23.2%)		158 (44.4%)		246 (33.5%)
**Crow's feet**						

Small		324 (85.5%)		291 (81.7%)		615 (83.7%)
Deep		55 (14.5%)		65 (18.3%)		120 (16.3%)
**Lip wrinkles**						
Small		358 (94.5%)		354 (99.4%)		712 (96.9%)
Deep		21 (5.5%)		2 (0.6%)		23 (3.1%)
**Nasolabial folds**						
Small		297 (78.4%)		273 (76.7%)		570 (77.6%)
Deep		82 (21.6%)		83 (23.3%)		165 (22.4%)
**Eyelid drooping**						
Small		272 (71.8%)		232 (65.2%)		504 (68.6%)
Deep		107 (28.2%)		124 (34.8%)		231 (31.4%)
**Skin color (Fitzpatrick)**						
Light		334 (88.1%)		326 (91.6%)		660 (89.8%)
Dark		45 (11.9%)		30 (8.4%)		75 (10.2%)
**Pigmentation Spots**						
0 None		17 (4.5%)		9 (2.5%)		26 (3.5%)
1 A few		336 (88.7%)		331 (93.0%)		667 (90.7%)
2 Many		26 (6.9%)		16 (4.5%)		42 (5.7%)
**Telangiectasias**						
0 None		205 (54.1%)		169 (47.5%)		374 (50.9%)
1 A few		140 (36.9%)		136 (38.2%)		276 (37.6%)
2 Many		34 (9.0%)		51 (14.3%)		85 (11.6%)
**Lip height**						
Mean (SD)		1.4 (0.4)		1.3 (0.4)		1.4 (0.4)
Median (Q1, Q3)		1.4 (1.2, 1.7)		1.3 (1.0, 1.5)		1.4 (1.1, 1.6)
Missing		3 (0.8%)		2 (0.6%)		5 (0.7%)
**Facial height**						
Mean (SD)		11.9 (0.9)		12.4 (1.4)		12.2 (1.2)
Median (Q1, Q3)		12.0 (11.5, 12.5)		12.7 (11.5, 13.3)		12.3 (11.5, 12.9)
Missing		3 (0.8%)		2 (0.6%)		5 (0.7%)

Supplementary Table 4. The association between multiple facial skin aging traits and lifestyle factors, adjusted for age and sex.

Variable	Wrinkle area		Perceived Facial Age		Glogau photoaging			Nasolabial folds			Drooping eyelids		
	Std.B	p	Std.B	p	OR	95% CI	p	OR	95% CI	p	OR	95% CI	p
Age	**0.89**	**7.48e-245**	**0.93**	**2.82E-271**	**1.25**	**1.21, 1.30**	**1.31e-37**	**1.10**	**1.08, 1.11**	**7.14e-31**	1.10	1.08, 1.11	1.08e-35
Sex (Male/Female)	**0.10**	**7.60e-09**	**0.12**	**1.03E-14**	**3.29**	**1.87, 5.96**	**5.17e-05**	1.33	0.89, 2.00	0.16	**1.87**	**1.27, 2.75**	**0.002**
Education													
University degree/no	0.01	0.37	**-0.06**	**2.32E-04**	**0.38**	**0.21, 0.67**	**0.0009**	0.73	0.49, 1.11	0.14	**0.54**	**0.37, 0.80**	**0.002**
Socioeconomic status (SES)													
High/Medium vs. Low	0.004	0.78	0.003	0.86	**0.29**	**0.09, 0.94**	**0.04**	0.75	0.30, 1.94	0.54	**0.38**	**0.15, 0.94**	**0.04**
Residence													
City<100K citizens / Village	-0.01	0.51	0.006	0.78	1	0.42, 2.41	0.10	1.32	0.66, 2.66	0.44	1.09	0.59, 2.01	0.77
City>100K citizens / Village	-0.0004	0.98	-0.02	0.25	**0.43**	**0.20, 0.90**	**0.03**	1.03	0.59, 1.84	0.92	**0.45**	**0.27, 0.74**	**0.002**
Type of job													
SM/Manual	0.002	0.94	-0.04	0.17	**0.58**	**0.15, 0.78**	**0.01**	1.28	0.68, 2.48	0.45	**0.50**	**0.27, 0.91**	**0.02**
LSM/Manual	0.01	0.65	**-0.08**	**0.002**	0.72	0.20, 0.99	0.05	0.88	0.46, 1.69	0.69	0.57	0.32, 1.00	0.05
Lifestyle factors													
Stress (Yes/No)	-0.031	0.09	**0.05**	**0.002**	**3.20**	**1.35, 7.57**	**0.008**	1.21	0.59, 2.48	0.60	**1.89**	**1.07, 3.33**	**0.03**
Yoga (Yes/No)	0.0007	0.97	-0.02	0.11	0.49	0.17, 1.33	0.18	1.07	0.51, 2.15	0.86	0.76	0.37, 1.49	0.43
PA (Active/Inactive)	0.02	0.29	-0.001	0.93	**0.50**	**0.28, 0.91**	**0.02**	0.74	0.47, 1.18	0.21	0.86	0.56, 1.35	0.52
Strengthening/Aerobic	0.03	0.26	**0.06**	**0.002**	1.14	4.46, 2.84	0.78	0.62	0.27, 1.33	0.24	0.83	0.43, 1.57	0.58
Yoga or balance/Aerobic	-0.002	0.92	-0.02	0.29	2.18	7.27, 6.60	0.16	2.42	1.03, 5.62	0.04	1.55	0.66, 3.61	0.31
Walk or Running (Yes/No)	0.002	0.94	-0.03	0.07	**0.37**	**1.57, 8.21**	**0.02**	0.75	0.40, 1.37	0.36	**0.53**	**0.29, 0.94**	**0.03**
Sleeping hours	-0.009	0.59	-0.02	0.27	0.97	0.77, 1.24	0.83	0.91	0.76, 1.08	0.26	1.08	0.92, 1.27	0.36
BMI	-0.003	0.87	0.01	0.54	1.05	1.00, 1.11	0.06	1.0	0.95, 1.04	0.82	**1.12**	**1.07, 1.17**	**4.01e-07**
Smoking													
Former smoker/Never	0.008	0.66	0.01	0.45	0.98	0.50, 1.89	0.94	1.32	0.81, 2.15	0.26	1.44	0.90, 2.28	0.12
Current smoker/Never	0.02	0.30	0.02	0.16	1.55	0.80, 3.01	0.19	1.40	0.83, 2.36	0.21	1.22	0.76, 1.95	0.41
Alcohol drink													
Occasionally/Never	0.002	0.92	0.006	0.78	1.44	0.59, 3.59	0.42	0.82	0.45, 1.50	0.51	1.21	0.67, 2.24	0.53
Frequently/Never	-0.01	0.62	-0.02	0.23	1.15	0.37, 3.53	0.81	0.88	0.39, 1.96	0.75	0.96	0.43, 2.10	0.91
Type of Diet													
Vegetarian/Varied	0.009	0.60	**-0.04**	**0.01**	0.73	0.11, 3.14	0.70	0.71	0.15, 2.30	0.61	1.10	0.36, 2.94	0.86
Protein/Varied	0.02	0.19	0.03	0.05	1.65	0.21, 8.62	0.58	NA	NA	NA	0.50	0.08, 1.91	0.37
Sugar intake													
Medium/low	**-0.08**	**0.004**	-0.02	0.34	1.07	0.39, 3.13	0.90	1.09	0.51, 2.47	0.84	**3.19**	**1.43, 7.83**	**0.007**
High/low	**-0.06**	**0.03**	**-0.05**	**0.02**	1.15	0.36, 3.88	0.81	1.08	0.45, 2.69	0.87	2.56	1.04, 6.81	0.05
Water per day	-0.002	0.92	-0.0009	0.95	1.27	0.58, 2.85	0.56	1.11	0.61,2.08	0.73	1.60	0.89,2.97	1.25
Cups of coffee per day													
Small/Non	**-0.05**	**0.02**	0.01	0.58	1.31	0.63, 2.76	0.48	0.92	0.54, 1.58	0.77	1.23	0.75, 2.03	0.42
Large/Non	0.0005	0.98	0.02	0.39	1.75	0.76, 4.09	0.19	0.86	0.45, 1.63	0.65	0.65	0.36, 1.17	0.15
Skin condition													
Skin color (Dark/Light)	**0.04**	**0.01**	**0.04**	**0.02**	0.58	0.22, 1.48	0.27	1.18	0.60, 2.24	0.63	0.92	0.48, 1.71	0.79
Tanning (Freq./ Rarely)	0.02	0.36	**0.04**	**0.009**	0.99	0.57, 1.70	0.96	**1.80**	**1.19, 2.75**	**0.006**	1.24	0.85, 1.83	0.26
Solarium													
Former solarium/Never	-0.03	0.15	0.02	0.19	1.34	0.67, 2.66	0.40	1.22	0.71, 2.08	0.46	1.16	0.73, 1.84	0.53
Current solarium/Never	-0.005	0.79	**0.05**	**0.002**	2.31	0.57, 8.40	0.22	**2.58**	**0.99, 6.19**	**0.04**	**0.10**	**0.006, 0.52**	**0.03**

Variable	Photonumeric full-face wrinkle score			Forehead wrinkles			Crow's feet			Lip wrinkles		
	OR	95% CI	p	OR	95% CI	p	OR	95% CI	p	OR	95% CI	p
Age	**1.14**	**1.10, 1.20**	**7.91e-09**	**1.07**	**1.05, 1.08**	**2.89e-23**	**1.09**	**1.07, 1.11**	**1.18e-24**	**1.21**	**1.14, 1.29**	**4.08e-09**
Sex (Male/Female)	0.77	0.30, 1.95	0.59	**3.77**	**2.64, 5.44**	**6.36e-13**	**1.57**	**1.01, 2.46**	**0.044**	**0.04**	**0.01, 0.18**	**1.50e-04**
Education												
University degree/no	1.09	0.43, 2.86	0.85	0.90	0.63, 1.29	0.55	0.80	0.51, 1.25	0.32	1.24	0.45, 3.51	0.67
Socioeconomic status (SES)												
High/Medium vs. Low	0.68	0.15, 4.3	0.65	1.07	0.45, 2.64	0.89	0.82	0.32, 2.33	0.69	1.46	0.27, 11.88	0.68
Residence												
City<100K/ Village	6.67	0.96, 134	0.10	0.79	0.43, 1.44	0.44	0.95	0.47, 1.94	0.90	0.22	0.01, 2.04	0.24
City>100K/ Village	2.85	0.53, 52.9	0.32	0.92	0.57, 1.48	0.72	**0.44**	**0.24, 0.79**	**0.006**	0.67	0.19, 2.71	0.54
Type of job												
SM/Manual	0.72	0.08, 6.86	0.76	0.81	0.48, 1.39	0.44	0.76	0.36, 1.64	0.48	0.96	0.08, 22.7	0.98
LSM/Manual	1.03	0.17, 8.40	0.97	0.90	0.55, 1.49	0.69	1.07	0.53, 2.24	0.84	1.79	0.22, 39.0	0.63
Lifestyle factor												
Stress (Yes/No)	NA	NA	NA	0.79	0.45, 1.37	0.40	**3.04**	**1.47, 6.29**	**0.003**	NA	NA	NA
Yoga (Yes/No)	1.06e-07	1.81e-147, 2.17	0.99	0.77	0.39, 1.46	0.44	**0.30**	**0.08, 0.81**	**0.03**	0.16	0.01, 1.11	0.12
PA (Yes/No)	1.15	0.44, 3.44	0.78	0.77	0.50, 1.17	0.22	0.73	0.45, 1.20	0.20	0.57	0.20, 1.65	0.29
Strengthening/Aerobic	0.91	0.04, 6.63	0.94	1.45	0.87, 2.42	0.16	0.55	0.20, 1.30	0.19	1.95	0.22, 14.01	0.51
Yoga or balance/Aerobic	1.66	2.21, 8.59	0.57	1.06	0.43, 2.48	0.89	0.88	0.32, 2.27	0.80	2.12	0.35, 11.51	0.39
Walk or Running (Yes/No)	0.50	0.10, 1.96	0.35	**0.59**	**0.36, 0.96**	**0.04**	**0.29**	**0.13, 0.62**	**0.002**	0.28	0.03, 1.52	0.18
Sleeping hours	1.12	0.79, 1.59	0.51	0.90	0.77, 1.05	0.18	1.02	0.85, 1.22	0.85	0.73	0.48, 1.07	0.12
BMI	0.92	0.80, 1.04	0.20	1.04	1.00, 1.08	0.07	1.04	0.99, 1.09	0.10	0.85	0.72, 0.99	0.05
Smoking												
Former smoker/Never	**4.13**	**1.41, 13.3**	**0.01**	0.91	0.58, 1.40	0.66	0.97	0.57, 1.64	0.92	2.23	0.67, 7.57	0.19
Current smoker/Never	**6.30**	**1.55, 26.1**	**0.009**	0.81	0.52, 1.25	0.34	1.07	0.58, 1.92	0.81	**4.75**	**1.14, 20.0**	**0.03**
Alcohol drink												
Occasionally/Never	1.10	0.37, 3.75	0.87	0.67	0.39, 1.14	0.14	1.48	0.76, 2.98	0.26	3.44	0.89, 17.8	0.10
Frequently/Never	0.70	0.03, 5.28	0.76	0.82	0.40, 1.66	0.58	1.12	0.43, 2.84	0.81	6.97	0.64, 68.6	0.09
Type of Diet												
Vegetarian/ Varied	2.46e-7	2.38e-260, 4.35	0.99	1.03	0.35, 2.73	0.95	0.72	0.11, 2.71	0.67	NA	NA	NA
Protein/ Varied	1.24e-06	1.72e-295, 1.80	0.99	1.17	0.39, 3.14	0.77	NA	NA	NA	NA	NA	NA
Sugar intake												
Medium/ Low	1.20	0.21, 22.8	0.86	0.73	0.40, 1.37	0.32	2.27	0.89, 7.07	0.12	0.53	0.10, 4.05	0.48
High/ Low	1.20	0.17, 24.3	0.88	0.69	0.33, 1.43	0.31	1.29	0.44, 4.42	0.66	0.09	0.01, 0.99	0.04
Water per day	0.52	0.19, 1.63	0.23	0.92	0.54, 1.60	0.77	1.01	0.54, 1.97	0.98	0.69	0.21, 2.55	0.55
Cups of coffee per day												
Small/Non	1.22	0.40, 4.58	0.75	1.46	0.91, 2.37	0.12	0.72	0.41, 1.29	0.26	1.87	0.43, 13.4	0.46
Large/Non	0.30	0.01, 2.31	0.31	1.13	0.66, 1.95	0.65	1.02	0.52, 2.02	0.95	3.14	0.48, 27.8	0.25
Skin condition												
Skin color (Dark/Light)	1.10	0.23, 3.81	0.90	1.15	0.64, 2.03	0.63	0.64	0.28, 1.37	0.28	0.61	0.08, 2.70	0.56
Tanning (Freq./ Rarely)	0.70	0.23, 1.90	0.50	1.06	0.75, 1.51	0.74	1.04	0.66, 1.64	0.87	0.86	0.24, 2.70	0.80
Solarium												
Former solarium/ Never	1.13	0.16, 4.91	0.88	1.25	0.80, 1.94	0.32	1.10	0.58, 1.99	0.77	0.43	0.02, 2.64	0.44
Current solarium/ Never	NA	NA	NA	1.94	0.87, 4.12	0.09	1.07	0.24, 3.33	0.92	NA	NA	NA

**Variable**	**Subjective aging rate**			**Pigmentation spots**			**Telangiectasias**			**Facial height**		**Lip height**	
	**OR**	**95% CI**	**p**	**OR**	**95% CI**	**p**	**OR**	**95% CI**	**p**	**Std.B**	**p**	**Std.B**	**p**
**Age**	0.99	0.98, 1.01	0.33	**1.08**	**1.04, 1.12**	**1.0e-4**	**1.06**	**1.04, 1.07**	**1.53e-19**	**0.24**	**6.94e-11**	-0.33	1.36e-20
**Sex (Male/Female)**	1.34	1.00, 1.81	0.05	**2.44**	**1.07, 5.96**	**0.04**	**1.59**	**1.16, 2.19**	**3.83e-03**	**0.22**	**2.04e-09**	**-0.22**	**4.33e-10**
**Education**													
**University degree/no**	**0.64**	**0.46, 0.89**	**0.007**	0.53	0.17, 1.36	0.22	0.84	0.61, 1.17	0.31	**0.12**	**9.0e-04**	**0.08**	**0.02**
**Socioeconomic status (SES)**													
**High/Medium vs. Low**	**0.27**	**0.13, 0.56**	**4.19e-04**	0.60	0.095, 2.09	0.49	0.64	0.31, 1.31	0.22	0.04	0.25	0.04	0.24
**Residence**													
**City<100K/ Village**	**0.50**	**0.28, 0.87**	**1.46e-02**	0.93	0.17, 5.21	0.93	0.88	0.52, 1.50	0.64	-0.005	0.92	-0.01	0.76
**City>100K/ Village**	**0.36**	**0.23, 0.57**	**1.77e-05**	0.55	0.13, 1.68	0.35	0.89	0.59, 1.36	0.60	0.06	0.18	0.04	0.38
**Type of job**													
**SM/Manual**	**0.48**	**0.30, 0.78**	**0.003**	0.35	0.09, 1.11	0.10	**0.58**	**0.35, 0.95**	**0.03**	0.05	0.67	0.07	0.18
**LSM/Manual**	0.64	0.40, 1.02	0.06	1.30	0.31, 4.77	0.70	0.80	0.51, 1.26	0.34	**0.19**	**0.0002**	0.08	0.10
**Lifestyle factor**													
**Stress (Yes/No)**	1.55	0.96, 2.53	0.08	NA	NA	NA	1.11	0.70, 1.77	0.66	**-0.46**	**1.72e-37**	**-0.23**	**3.78e-10**
**Yoga (Yes/No)**	**0.33**	**0.19, 0.55**	**4.30e-05**	0.98	0.31, 4.33	0.97	1.01	0.58, 1.73	0.98	0.04	0.31	0.05	0.20
**Physical activity (Yes/No)**	**0.61**	**0.41, 0.89**	**0.01**	2.09	0.77, 5.17	0.13	**0.60**	**0.40, 0.89**	**0.01**	-0.004	0.91	-0.05	0.18
**Strengthening/Aerobic**	0.71	0.46, 1.09	0.11	**6.65**	**1.75, 43.68**	**0.02**	0.77	0.49, 1.21	0.26	0.28	7.84e-09	0.02	0.34
**Yoga or balance/Aerobic**	0.74	0.37, 1.46	0.38	1.71	0.28, 32.95	0.63	1.18	0.57, 2.43	0.66	0.08	0.07	0.01	0.82
**Walk or Running (Yes/No)**	1.31	0.88, 1.98	0.19	1.07	0.35, 3.96	0.91	0.67	0.43, 1.02	0.07	**-0.17**	**0.0002**	-0.03	0.56
**Sleeping hours**	0.88	0.77, 1.00	0.05	**1.56**	**1.09, 2.24**	**0.01**	0.88	0.77, 1.01	0.07	-0.01	0.71	0.06	0.09
**BMI**	**0.11**	**1.07, 1.15**	**2.80e-07**	0.98	0.89, 1.08	0.65	**1.09**	**1.05, 1.14**	**7.74e-06**	0.05	0.21	-0.01	0.74
**Smoking**													
**Former smoker/Never**	0.95	0.65, 1.39	0.79	**0.27**	**0.09, 0.80**	**0.02**	1.26	0.84, 1.90	0.26	-0.07	0.08	-0.03	0.39
**Current smoker/Never**	1.44	0.99, 2.12	0.06	**0.31**	**0.11, 0.80**	**0.02**	1.11	0.75, 1.63	0.60	-0.03	0.37	0.03	0.42
**Alcohol drink**													
**Occasionally/ Never**	1.12	0.70, 1.79	0.63	0.80	0.12, 2.92	0.77	0.70	0.42, 1.16	0.17	-0.03	0.55	0.007	0.88
**Frequently/ Never**	0.89	0.48, 1.66	0.72	0.44	0.06, 2.38	0.36	**0.47**	**0.24, 0.92**	**0.03**	0.02	0.69	0.05	0.29
**Type of Diet**													
**Vegetarian/ Varied**	**0.29**	**0.11, 0.70**	**0.008**	0.87	0.16, 16.14	0.89	2.05	0.86, 5.14	0.11	0.05	0.18	0.05	0.12
**Protein/ Varied**	0.55	0.22, 1.37	0.19	NA	NA	NA	1.29	0.50, 3.25	0.60	0.03	0.48	0.05	0.16
**Sugar intake**													
**Medium/ Low**	0.94	0.53, 1.63	0.82	2.50e-07	2.74e-108, 3e+11	0.99	0.77	0.43, 1.36	0.37	-0.05	0.39	-0.02	0.78
**High/ Low**	0.95	0.49, 1.81	0.89	NA	NA	NA	0.75	0.38, 1.45	0.39	-0.04	0.52	0.06	0.30
**Water per day (>2/<2)**	0.84	0.52, 1.34	0.46	1.65	0.46, 4.69	0.39	1.60	0.97, 2.66	0.07	0.001	0.97	0.02	0.50
**Cups of coffee per day**													
**Small/Non**	0.92	0.62, 1.37	0.69	0.82	0.22, 2.38	0.73	1.17	0.77, 1.78	0.48	0.07	0.14	-0.06	0.20
**Large/Non**	1.28	0.80, 2.03	0.31	0.72	0.18, 2.51	0.62	1.17	0.72, 1.88	0.53	0.04	0.39	0.009	0.84
**Skin condition**													
**Skin color (Dark/Light)**	0.71	0.44, 1.16	0.17	1.46	0.41, 9.34	0.62	**0.46**	**0.26, 0.79**	**5.16e-03**	0.03	0.35	0.02	0.56
**Tanning (Freq./ Rarely)**	0.80	0.59, 1.08	0.15	2.01	0.88, 5.03	0.11	1.05	0.77, 1.44	0.77	-0.02	0.63	-0.002	0.96
**Solarium**													
**Former solarium/ Never**	0.76	0.53, 1.11	0.15	0.87	0.38, 2.10	0.75	1.40	0.96, 2.04	0.08	0.03	0.37	0.06	0.09
**Current solarium/ Never**	**0.39**	**0.20, 0.75**	**0.005**	NA	NA	NA	0.62	0.28, 1.28	0.21	**0.09**	**0.01**	0.05	0.18

Glogau photogaing: a two-stage categorization was used, comparing categories III and IV vs. categories I and II (reference); Nasolabial folds, eyelid drooping, photonumeric full-face wrinkles, forehead wrinkles, crow's feet, lip wrinkles: deep wrinkles vs. small wrinkles (reference) were analysed; subjective aging rate was measured on a 3-point scale: 0 (younger than real age), 1 (as real age) and 2 (older than real age); wrinkle area, facial perceived age, facial height and lip height were analysed on a continuous scale.

Type of job: Sedentary mental (SM), Long hours Sedentary Mental (LSM), Manual (M); Cups of coffee per day: small (1-2 cups/day), large (≥3 cups/day); Stress vulnerability: measured on a 3-point scale: low, medium, high

Std.B: the standardized beta regression coefficient of the respective variable from the linear regression model applied for continuous traits; OR: Odds ratio from the logistic regression applied for categorical traits, 95% CI: confidence interval. p: significant p-values (<0.05) are marked with bold. Due to the small number of observations in some categories, the results were not estimated (NA) because of instability in the model's results. **Results of association were adjusted for age and sex**.

Supplementary Table 9. Associations between selected facial skin aging traits, including photonumeric wrinkle classifications, Glogau photoaging, nasolabial folds, and eyelid drooping, facial height and lip height with various measures of epigenetic aging.

	**Photonumeric full-face wrinkle score N=735**			**Forehead wrinkles N=735**			**Crow's feet N=735**			**Lip wrinkles N=735**		
	**OR**	**95% CI**	**p**	**OR**	**95% CI**	**p**	**OR**	**95% CI**	**p**	**OR**	**95% CI**	**p**
**Model 1 (age and sex adjusted)**												
DNAm age Horvath	1.02	0.92, 1.12	0.70	0.98	0.94, 1.02	0.44	0.98	0.93, 1.03	0.43	0.98	0.87, 1.11	0.80
DNAm age Hannum	0.97	0.87, 1.08	0.55	1.02	0.97, 1.07	0.49	1.02	0.96, 1.08	0.58	0.96	0.85, 1.09	0.53
DNAm age Skin&Blood	1.05	0.92, 1.19	0.45	1.02	0.96, 1.07	0.58	1.07	1.00, 1.15	0.05	0.88	0.75, 1.01	0.07
IEAA	1.03	0.93, 1.14	0.53	0.99	0.95, 1.03	0.57	0.98	0.93, 1.03	0.42	0.97	0.86, 1.10	0.64
EEAA	0.97	0.89, 1.06	0.55	1.01	0.98, 1.05	0.49	1.01	0.96, 1.05	0.77	0.98	0.89, 1.08	0.66
PhenoAgeAccel	1.03	0.95, 1.12	0.42	0.98	0.95, 1.01	0.26	1.01	0.97, 1.05	0.57	1.04	0.95, 1.14	0.37
GrimAgeAccel	1.06	0.94, 1.18	0.29	1.00	0.95, 1.05	0.91	1.03	0.97, 1.09	0.31	**1.13**	**1.01, 1.26**	**0.03**
FitAgeAccel	1.07	0.96, 1.19	0.23	1.03	0.98, 1.08	0.26	0.99	0.93, 1.04	0.61	1.07	0.96, 1.20	0.22
PACE	10.10	0.10, 891.04	0.31	2.36	0.42, 13.27	0.33	4.02	0.45, 35.76	0.21	NA	NA	NA
PoAm	0.24	0.0002, 176.81	0.68	2.75	0.24, 31.87	0.42	10.51	0.47, 229.90	0.14	NA	NA	NA
Zhang MRS	0.67	0.16, 2.60	0.57	1.28	0.75, 2.18	0.37	0.93	0.47, 1.83	0.84	1.90	0.47, 7.67	0.36
DNAmTL	0.30	0.02, 3.81	0.36	0.88	0.31, 2.52	0.81	0.59	0.16, 2.12	0.42	0.04	0.002, 0.79	0.04
**Model 2 (adjusted for age, sex, lifestyle and blood cell proportion)**												
IEAA	1.02	0.91, 1.14	0.70	0.99	0.95, 1.03	0.64	0.97	0.92, 1.03	0.31	0.96	0.83, 1.09	0.55
EEAA	0.99	0.89, 1.10	0.83	1.02	0.98, 1.07	0.32	1.02	0.97, 1.08	0.50	0.94	0.82, 1.07	0.38
Skin&BloodAgeAccel	1.03	0.89, 1.19	0.67	1.02	0.97, 1.08	0.43	**1.08**	**1.00, 1.16**	**0.04**	**0.81**	**0.67, 0.96**	**0.02**
PhenoAgeAccel	1.04	0.96, 1.14	0.33	0.97	0.94, 1.01	0.15	1.02	0.98, 1.07	0.35	1.06	0.96, 1.18	0.28
GrimAgeAccel	1.01	0.88, 1.15	0.91	1.00	0.95, 1.07	0.89	1.07	0.99, 1.15	0.07	1.09	0.94, 1.27	0.26
FitAgeAccel	1.02	0.90, 1.16	0.76	1.05	0.99, 1.10	0.08	0.99	0.93, 1.06	0.79	1.01	0.88, 1.16	0.86
PACE	8.23	0.04, 1589.37	0.44	2.29	0.36, 14.80	0.38	6.64	0.60, 74.12	0.12	NA	NA	NA
PoAm	0.01	6.9e-07, 24.00	0.24	2.94	0.17, 53.30	0.46	42.44	0.98, 1964.22	0.05	NA	NA	NA
Zhang MRS	0.68	0.08, 5.63	0.73	1.34	0.59, 3.05	0.49	1.47	0.52, 4.19	0.47	3.39	0.29, 40.98	0.33
DNAmTL	0.53	0.03, 10.29	0.68	0.70	0.22, 2.23	0.54	0.47	0.11, 1.94	0.30	0.05	0.001, 1.53	0.10

	Facial height N=730		Lip height N=730		Glogau photoaging N=735			Nasolabial folds N=735			Eyelid drooping N=735		
	**Std.B**	**P**	**Std.B**	**p**	**OR**	**95% CI**	**p**	**OR**	**95% CI**	**p**	**OR**	**95% CI**	**p**
**Model 1 (age and sex adjusted)**													
DNAm age Horvath	-0.05	0.62	-0.03	0.77	0.97	0.91,1.03	0.34	0.98	0.94, 1.03	0.39	1.00	0.96, 1.05	0.86
DNAm age Hannum	-0.08	0.51	0.01	0.96	1.01	0.94,1.08	0.81	1.02	0.96, 1.07	0.54	0.99	0.94, 1.04	0.67
DNAm age Skin&Blood	0.13	0.44	0.09	0.57	0.93	0.85,1.02	0.15	1.00	0.94, 1.07	0.92	1.00	0.95, 1.07	0.89
IEAA	0.01	0.78	0.00	0.97	0.95	0.89,1.02	0.16	0.97	0.92, 1.01	0.17	1.00	0.95, 1.04	0.85
EEAA	-0.02	0.54	-0.01	0.80	1.02	0.96, 1.08	0.55	1.02	0.97, 1.06	0.45	1.00	0.96, 1.04	0.91
PhenoAgeAccel	0.02	0.49	0.02	0.52	1	0.94, 1.05	0.92	1.00	0.96, 1.04	0.84	1.00	0.97, 1.04	0.84
GrimAgeAccel	0.05	0.20	0.02	0.62	**1.08**	**1.01, 1.16**	**0.03**	0.99	0.94, 1.05	0.80	1.01	0.96, 1.06	0.69
FitAgeAccel	0.00	0.94	0.02	0.65	1.02	0.96,1.10	0.49	1.02	0.97, 1.07	0.51	0.99	0.94, 1.04	0.69
PACE	-0.01	0.80	-0.05	0.17	8.8	0.69,117.24	0.10	0.38	0.05, 2.80	0.34	3.06	0.48, 19.7	0.24
PoAm	0.03	0.35	-0.03	0.34	NA	NA	NA	1.63	0.09, 27.9	0.74	7.85	0.56, 113	0.13
Zhang MRS	0.00	0.91	-0.01	0.85	1.98	0.86,4.60	0.11	1.19	0.64, 2.22	0.58	0.88	0.49, 1.58	0.68
DNAmTL	0.10	0.17	-0.03	0.68	0.29	0.05,1.49	0.14	0.31	0.09, 1.02	0.05	1.31	0.42, 4.10	0.65
**Model 2 (adjusted for age, sex, lifestyle and blood cell proportion)**													
IEAA	0.04	0.16	0.01	0.75	0.96	0.90, 1.03	0.28	0.96	0.91, 1.01	0.11	0.99	0.94, 1.04	0.63
EEAA	-0.02	0.66	0.00	0.94	1.03	0.96, 1.11	0.36	1.01	0.96, 1.06	0.72	0.98	0.93, 1.03	0.36
Skin&BloodAgeAccel	0.05	0.08	0.03	0.43	0.93	0.85, 1.03	0.16	0.99	0.93, 1.06	0.74	0.99	0.93, 1.06	0.81
PhenoAgeAccel	0.01	0.70	0.03	0.43	1.00	0.94, 1.06	0.96	1.00	0.96, 1.04	0.92	1.00	0.96, 1.04	0.94
GrimAgeAccel	0.04	0.29	-0.01	0.81	1.09	1.00, 1.20	0.05	0.99	0.93, 1.06	0.80	1.01	0.95, 1.08	0.72
FitAgeAccel	0.02	0.55	0.01	0.79	1.02	0.94, 1.11	0.66	1.01	0.95, 1.07	0.83	0.97	0.92, 1.03	0.28
PACE	-0.02	0.50	-0.06	0.12	9.45	0.53, 181	0.13	0.45	0.05, 4.07	0.48	3.88	0.50, 30.4	0.19
PoAm	0.01	0.77	-0.06	0.14	NA	NA	NA	2.27	0.08, 68.8	0.64	21.2	0.84, 573	0.07
Zhang MRS	-0.04	0.40	-0.07	0.24	3.14	0.83, 12.3	0.10	1.45	0.56, 3.74	0.44	0.63	0.25, 1.55	0.31
DNAmTL	0.05	0.47	-0.01	0.84	0.37	0.06, 2.44	0.30	0.41	0.11, 1.53	0.18	1.68	0.48, 5.90	0.42

The analysis included selected epigenetic clocks, namely DNAm Age Horvath2013, DNAm Age Hannum, DNAm Age Skin & Blood and DNAmTL. Additionally, epigenetic age acceleration (EAA) measures were calculated as the residuals from regressing DNAm age on chronological age. EAA measures comprised intrinsic epigenetic age acceleration (IEAA), calculated using the Horvath2013 clock; extrinsic epigenetic age acceleration (EEAA), estimated using the Hannum clock; DNAm PhenoAge acceleration (PhenoAgeAccel); DNAm GrimAge acceleration (GrimAgeAccel); and DNAm FitAge acceleration (FitAgeAccel). Additionally, DNAm-based pace of aging metrics (PACE and PoAm) and the epigenetic mortality risk score (Zhang MRS) were included in the analysis.

Std. B: standardized beta coefficient from linear regression; OR: odds ratio from logistic regression; 95% CI: 95% confidence interval.

Model 2 for continuous traits: lm(wrinkle(linear) ~ EAA + Age + Sex + Smoking + Stress + Skin color Fitzpatrick + Solarium + Frequency of tanning + CD8T + CD4T + NK + Bcell + Mono + Gran)

Model 2 for binary traits: glm(wrinkle (binary) ~ EAA + Age + Sex + Smoking + Stress + Skin color Fitzpatrick + Solarium + Frequency of tanning + CD8T + CD4T +NK +Bcell +Mono +Gran)

Significant results are marked with bold

Facial height and lip height were measured on a continous scale. Glogau photogaing: a two-stage categorization was used, comparing categories III and IV vs. categories I and II (reference); Nasolabial folds, eyelid drooping, full face wrinkles, forehead wrinkles, crow's feet, wrinkles over the lips: deep wrinkles vs. small wrinkles (reference) were analysed.

Supplementary Table 11. The list of the top GO terms for DMSs associated with different facial skin aging phenotypes at suggestive $p < 1.0\times10^{-5}$.

Skin aging trait	Ontology	TERM	Sig. Genes in Set	P.DE
Wrinkle area	CC	apical part of cell	EDAR,NRG1,GNAT3	0.0004
	BP	positive regulation of peptidyl-tyrosine autophosphorylation	NRG1	0.0007
	BP	signaling	EDAR,RIMBP2,NRG1,GNAT3,WNT9A,DLGAP1,NFE2L3	0.0008
	BP	cell communication	EDAR,RIMBP2,NRG1,GNAT3,WNT9A,DLGAP1,NFE2L3	0.0008
	BP	regulation of peptidyl-tyrosine autophosphorylation	NRG1	0.001
	CC	synapse	AGAP1,RIMBP2,NRG1,DLGAP1	0.001
	BP	tube lumen cavitation	EDAR	0.001
	BP	salivary gland cavitation	EDAR	0.001
	BP	ERBB2-ERBB4 signaling pathway	NRG1	0.002
	MF	ErbB-3 class receptor binding	NRG1	0.002
Photonumeric full-face wrinkle score	BP	generation of neurons	CDH4,CNGB1,EML1,ZMYND8,GLI3,GLDN,NGF,ROR1,ATL1,LRRC7,BCL11B,SOX2,TBCD	0.0002
	BP	nervous system development	CDH4,CNGB1,C1QL2,EML1,ZMYND8,SDCBP2,GLI3,GLDN,NGF,ROR1,ATL1,LRRC7,BCL11B,SIM1,SLC6A3,SOX2,TBCD	0.0003
	BP	neuron differentiation	CDH4,CNGB1,ZMYND8,GLI3,GLDN,NGF,ROR1,ATL1,LRRC7,BCL11B,SOX2,TBCD	0.0004
	BP	adenohypophysis development	SLC6A3,SOX2	0.0009
	BP	thymocyte apoptotic process	GLI3,BCL11B	0.002
	CC	axon	CNGB1,GLDN,NGF,ROR1,ATL1,LRRC7,SLC6A3	0.002
	BP	regulation of neuron differentiation	GLI3,NGF,BCL11B,SOX2	0.002
	BP	subpallium development	GLI3,BCL11B	0.002
	BP	regulation of collagen binding	GTPBP4	0.003
	BP	negative regulation of collagen binding	GTPBP4	0.003
Lip wrinkles	BP	spinal cord interneuron axon guidance	EVX1,LHX9	6.95e-05
	BP	interneuron axon guidance	EVX1,LHX9	0.0001
	BP	regulation of fermentation	CAVIN3,ACTN3	0.0002
	BP	membrane depolarization during SA node cell action potential	HCN4,CACNA1D	0.0002
	BP	fermentation	CAVIN3,ACTN3	0.0003
	BP	negative regulation of feeding behavior	MC4R,UCN	0.0003
	CC	neuron projection	HCN4,EVX1,SIRT2,GABRA1,KCND2,KCNQ2,PCSK2,SYBU,CX3CL1,TULP1,UCN,CALCR,CAMK2A,ESPN,PSD2,RNF40	0.0004
	MF	voltage-gated monoatomic cation channel activity	HCN4,KCND2,KCNQ2,CACNA1D,CACNB4	0.0005
	CC	postsynaptic specialization	DMTN,GABRA1,KCND2,RPL38,PRRT1,CAMK2A,DCLK1,DLGAP1	0.0005
	BP	peptidyl-threonine phosphorylation	DMTN,SIRT2,HIPK2,CAMK2A	0.0005
Perceived facial age	MF	RNA polymerase I cis-regulatory region sequence-specific DNA binding	UBTF	0.002
	BP	negative regulation of microtubule nucleation	ARHGEF7	0.003
	MF	N-acetyllactosaminide beta-1,6-N-acetylglucosaminyltransferase activity	GCNT2	0.003
	MF	POZ domain binding	KLHL17	0.003
	MF	RNA polymerase I general transcription initiation factor activity	UBTF	0.004
	BP	zonula adherens assembly	DLG5	0.005
	BP	regulation protein catabolic process at postsynapse	KLHL17	0.006
	MF	tRNA (guanine) methyltransferase activity	TARBP1	0.007
	BP	regulation of smoothened signaling pathway	GAS1,DLG5	0.008
	MF	insulin-like growth factor receptor activity	CRIM1	0.009

P.DE (P-value for over-representation of the GO term). ONTOLOGY: testing GO pathways. "BP" - biological process, "CC" - cellular component, "MF" - molecular function. Sig. Genes in Set: Significant differentially methylated genes overlapping with the gene set of interest.

Supplementary Table 12. Functional analysis of the genome-wide significant SNPs detected in GWAS.

Skin aging trait	chr	variant	Genotype	Enhancer histone marks	Motifs changed	GENCODE genes
Wrinkle Area	18	rs73943403	A>G	ESC, BLD	Foxa, STAT, ZNF263	RP11-78F17.1
	18	rs79620305	A>G		4 altered motifs	RP11-78F17.1
Perceived Facial Age	19	rs74854711	G>A		SMC3	ZC3H4
	19	rs113125564	T>C	SKIN, GI, MUS	Eomes, Pax-4, Sox	ZC3H4
